# New Oral Anticoagulants for Venous Thromboembolism Prophylaxis in Total Hip and Knee Arthroplasty: A Systematic Review and Network Meta-Analysis

**DOI:** 10.3389/fphar.2021.775126

**Published:** 2022-01-17

**Authors:** Yi-hu Yi, Song Gong, Tian-lun Gong, Ling-yun Zhou, Can Hu, Wei-hua Xu

**Affiliations:** ^1^ Department of Orthopaedics, Union Hospital, Tongji Medical College, Huazhong University of Science and Technology, Wuhan, China; ^2^ Department of Pharmacy, Third Xiangya Hospital, Central South University, Changsha, China

**Keywords:** network meta-analysis, new oral anticoagulants, total knee arthroplasty, total hip arthroplasty, venous thromboembolism

## Abstract

**Background:** There is controversy over whether use of new oral anticoagulants (NOACs) associates with increased hemorrhage risk compared with non-NOAC. Meanwhile, determining which NOAC to use remains unclear. We aimed to summarize the evidence about NOACs in venous thromboembolism (VTE) prevention for patients with total hip and knee arthroplasty (THA and TKA).

**Methods:** We searched RCTs assessing NOACs for VTE prophylaxis in adults undergoing THA and TKA in Medline, Embase, and Cochrane up to May 2021. Primary outcomes were VTE [included deep vein thrombosis (DVT) and pulmonary embolism (PE)], major VTE, and major bleeding. The rank probabilities of each treatment were summarized by the surface under the cumulative ranking curve area (SUCRA).

**Results:** 25 RCTs with 42,994 patients were included. Compared with non-NOAC, NOACs were associated with a decreased risk of VTE (RR 0.68; 95% CI 0.55–0.84) and major VTE (RR = 0.52; 95% CI 0.35–0.76). Additionally, rivaroxaban, apixaban, and edoxaban but not dabigatran and betrixaban, did confer a higher efficacy compared with non-NOAC. None of the individual NOACs increased the risk of bleeding, while apixaban and betrixaban were even associated with a decreased risk of bleeding. In the comparison of different NOACs, rivaroxaban was associated with the greatest benefits in VTE (SUCRA = 79.6), DVT (SUCRA = 88.8), and major VTE (SUCRA = 89.9) prevention. Furthermore, subgroup analysis confirmed that NOACs associated with a higher efficacy tendency in patients with follow-up duration <60 days than follow-up duration ≥60 days.

**Conclusion:** Evidence suggests that NOACs exert more benefits on VTE prophylaxis, and none of the individual NOACs increased hemorrhage compared with non-NOAC. Among various NOACs, rivaroxaban is recommended in patients with lower bleeding risk, and apixaban is recommended in patients with higher bleeding risk.

**Systematic Review Registration**: [https://www.crd.york.ac.uk/prospero/], identifier [CRD42021266890].

## Introduction

Total hip arthroplasty (THA) and total knee arthroplasty (TKA) are common and effective prophylaxis for degenerative joint diseases, such as osteoarthritis (Learmonth et al., 2007). A more than 10% incidence of venous thromboembolism (VTE) [deep vein thrombosis (DVT) and pulmonary embolism (PE)] has been reported after knee or hip arthroplasty (Falck-Ytter et al., 2012). The PE caused by undiagnosed or untreated DVT has substantial health care costs, and seriously affects the functionality of patients and even contributes to a mortality rate of 70% among patients (Strandness et al., 1983; Xie et al., 2019).

Since the risk of VTE is higher in the THA and TKA patients, mechanical interventions such as anti-embolism stockings, foot impulse devices, and intermittent pneumatic compression devices were recommended to start on admission. Meanwhile, pharmacological interventions were also recommended to be added after arthroplasty (Lewis et al., 2019). Currently, anticoagulants for preventing VTE include simple oral agents (aspirin), vitamin K antagonists (warfarin), injectable agents [low-molecular-weight heparin (LMWH)], and novel oral anticoagulants (NOACs, including rivaroxaban, apixaban, edoxaban, dabigatran, and betrixaban) (Matharu et al., 2020). The traditional injectable agent LMWH is not convenient for patients to use after discharge, and the traditional oral anticoagulant warfarin is difficult to control in clinical application because it interacts with many drugs and has a narrow therapeutic window (Miranda et al., 2016). Meanwhile, considerable contention surrounds the use of aspirin due to the uncertainty around its efficacy in venous thromboembolism prevention (Lewis et al., 2019). The NOACs as a nonvitamin K antagonist oral anticoagulant, which does not require routine monitoring in clinical, represent a convenient alternative to conventional treatment for prophylaxis against VTE in arthroplasty patients (Steffel et al., 2018).

However, the extensive clinical application of NOACs has raised concerns on its efficacy and safety. Previous studies indicated that NOACs have obvious advantages in curative effect compared with other types of anticoagulants (Venker et al., 2017a), but there are conflicting results as to whether the new anticoagulants increase the risk of bleeding and which of the new anticoagulants show the highest efficacy in the hip and knee arthroplasty population. No head-to-head trial has directly addressed the effectiveness and safety of these therapies or has clearly defined the choice of a specific NOAC.

On this basis, a more accurate understanding of NOACs is required to make appropriate drug choices, both at an individual and public health level, informing drug prescribing and procurement. Our systematic review and network meta-analysis of randomized controlled trials (RCTs) were undertaken to compare the efficacy and safety of NOACs and provide a ranking of the anticoagulants in patients undergoing THA and TKA.

## Methods

The systemic review and network analysis was performed according to the recommendations from the Cochrane Handbook and the PRISMA extension statement for reporting of systematic reviews incorporating network meta-analyses of health care interventions. Formal ethical approval was not required. The study is registered with PROSPERO (CRD42021266890).

### Search Strategy

We restricted our analysis to studies that were phase III RCTs and met all of the following inclusion criteria: (1) the population was defined as adult patients (≥18 years) undergoing THA or TKA surgery admitted to and discharged from the hospital; (2) the included study should be an RCT involving one or more interventions including a specific NOAC (all types and doses) and the control group were unrestricted, trials using hybrid VTE prophylaxis strategies in which two agents used were included to reflect current practice; (3) studies reported the interest endpoints included incidence of VTE, DVT (symptomatic and asymptomatic), PE, major VTE (defined as the composite of proximal DVT, nonfatal pulmonary embolism, or death from venous thromboembolism) (Eriksson et al., 2008; Lassen et al., 2009), major bleeding, all bleeding, clinically relevant nonmajor bleeding, all cause death, ischemic stroke, and myocardial infarction. Exclusion criteria include: (1) No RCTs; (2) duplicates; (3) studies published in non-English; (4) full text could not be acquired online. For trials reporting more than one publication, data was extracted from the most complete publication. No ethical approval was required for this study.

We searched all relevant studies in Medline via the PubMed interface, Embase, and Cochrane from inception to May 20, 2021, with MeSH and keywords, relating to various combinations of the name of NOACs and population (THA or TKA). The search details are shown in Supplementary Table S1. Inclusion decisions were made by two reviewers (Can Hu and Yi-hu Yi) and quality checked by a senior reviewer (Wei-hua Xu). Any disagreement was resolved by discussion between the two reviewers or input from the guideline committee, or both. Two investigators screened the studies independently. In cases of inconsistency, a standardized predesigned data extraction form was used to obtain the relevant data from each RCT, including study design, baseline demographic characteristics, geographical location, numbers enrolled and randomized, allocation concealment, blinding, VTE prophylaxis regimens (including dosage and duration), outcomes of interest, and follow-up duration.

### Data Analysis

Two investigators (Can Hu and Yi-hu Yi) collected the data from reports independently. Data extracted from each study include research identifiers (research title, year of publication, first author, journal name, study characteristics); the baseline characteristics of patients (source of the patients—hospital and country, inclusion and exclusion criteria, sample size, the age and sex of patients); medication regimen (dosage form, dose, route of administration, duration of treatment); and outcomes data.

### Outcome Measures

The effectiveness of NOACs is reflected in preventing the occurrence of VTE events. In addition, the use of NOACs also faces the risk of hemorrhagic events and cardiovascular events, and these outcomes have been reported in previous studies (Sardar et al., 2015). Primary outcomes were VTE and major VTE, secondary outcomes were DVT, PE, and all cause of death. Major bleeding served as a primary safety outcome, secondary safety outcomes were all bleeding, clinically relevant nonmajor bleeding, and cardiovascular events (including ischemic stroke and myocardial infarction) outcomes.

### Risk-of-Bias Assessments

The methodological quality of the included studies was estimated based on the Cochrane Risk of Bias criteria. The seven items used to assess bias in each RCT included randomization sequence generation, allocation concealment, blinding of participants and personnel, blinding of outcome assessment, incomplete outcome data, selective reporting, and other source of biases. Each quality item was graded as low risk, high risk, or no clear risk. A funnel plot was generated to examine the potential publication bias if the number of included studies was more than 10 (Wei et al., 2018b).

### Statistical Analysis

Both direct pairwise meta-analysis and network meta-analysis were performed using the STATA statistical software (version 13.0, Stata Corp, College Station, TX) and Review Manager 5.3 (RevMan, The Cochrane Collaboration, Oxford, United Kingdom) (Zhou et al., 2018). The different treatment strategies were treated as separate nodes. Individual studies and pooled estimates were derived and presented in forest plots. Results were reported as risk ratios (RRs) with their 95% confidence intervals (CIs), significant differences were considered when the 95% CI of RR did not include 1. The between-study heterogeneity was evaluated through *I* ^*2*^ test and Q statistic. *I* ^*2*^ of >50% indicated considerable heterogeneity, and a *p-*value of <0.05 at Q statistic represented a significant heterogeneity (Higgins et al., 2003). The random effect model was used when I^2^ > 50% or *p* < 0.05, otherwise the fixed effect model was used (Wei et al., 2018a). Subgroup analysis based on individual NOACs (rivaroxaban, apixaban, edoxaban, dabigatran, and betrixaban) and patient characteristics (THA or TKA, follow up duration ≥60 days or <60 days) was used to explore possible causes of heterogeneity among study results. Sensitivity analyses were performed to identify the effect of a single trial by sequential elimination of each trial from the pool.

For network comparison between the different NOACs using non-NOAC as the reference comparator, and multivariate random-effect analysis was performed on a data set of point estimates. Node-splitting analysis was used to calculate the consistency of data, by comparing direct and indirect estimates. Surface under the cumulative ranking curve area (SUCRA) is a relative ranking measure based on cumulative probability plots, which accounts both for the location and the variance of all relative treatment effects (Tereshchenko et al., 2016). For each endpoint of all anticoagulants, we calculated the SUCRA to provide a hierarchy. SUCRA was used to rank the treatments, for which a larger value indicates higher rank.

## Results

### Study Search and Study Characteristics

**FIGURE 1:**
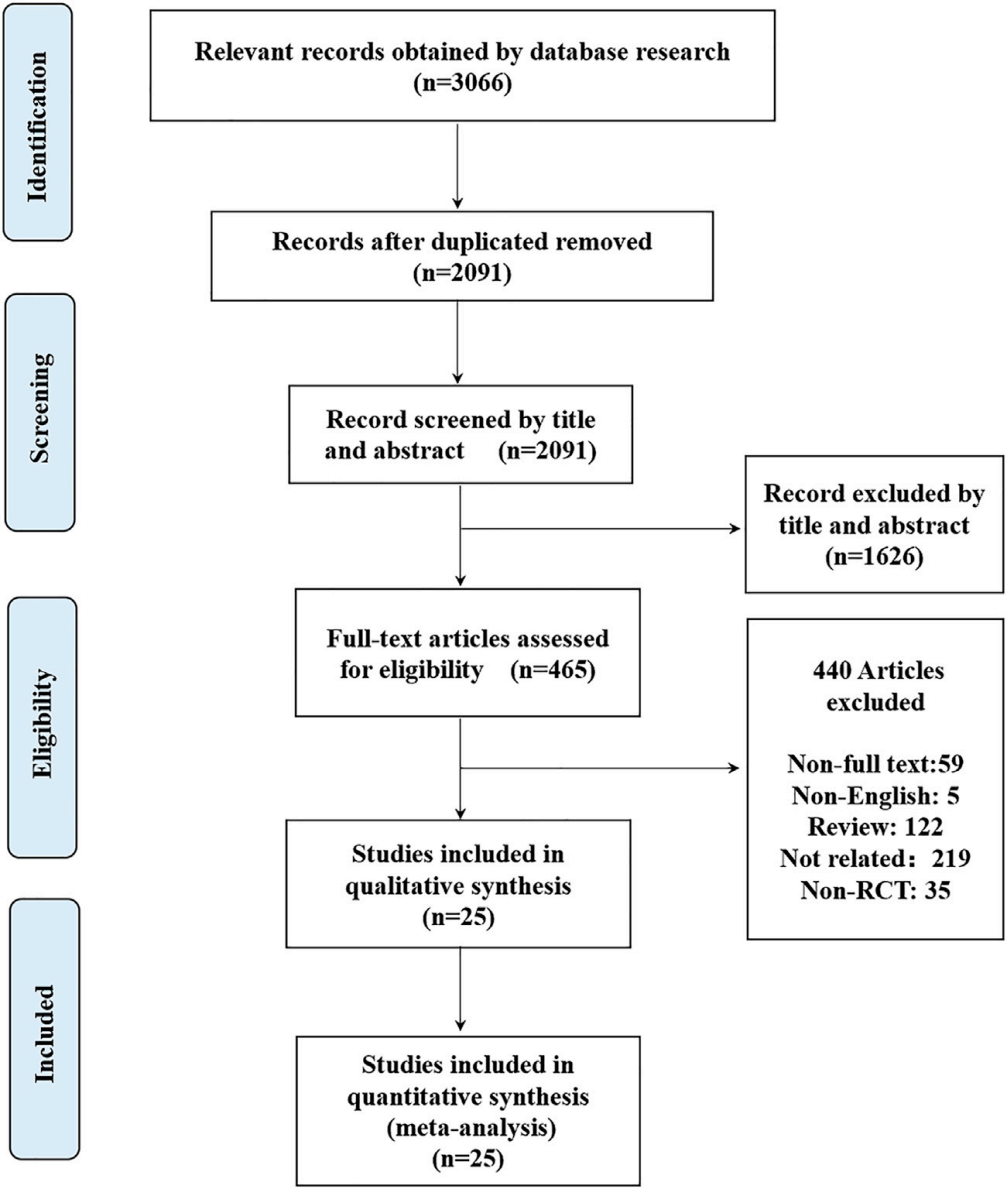
Flow diagram of study selection.

The initial search identified 3066 potentially relevant citations (Figure 1). After screening titles and abstracts, 465 articles remained for full-text assessment. After systematically reviewing the remaining 465 full texts, 440 articles were subsequently excluded, any NOAC phase II studies were not considered. Finally, 25 RCTs published up to May 20, 2021, were included in the network meta-analysis (Eriksson et al., 2008; Kakkar et al., 2008; Lassen et al., 2008; Turpie et al., 2009; Lassen et al., 2007; Lassen et al., 2009; Lassen et al., 2010; Lassen et al., 2010; Eriksson et al., 2007; Eriksson et al., 2011; Eriksson et al., 2007; Ginsberg et al., 2009; Fuji et al., 2010; Turpie et al., 2009; Anderson et al., 2018; Fuji et al., 2014; Fuji et al., 2015; Kim et al., 2016; Jiang et al., 2014; Zou et al., 2014; Verhamme et al., 2013; Jiang et al., 2018; Mirdamadi et al., 2014; Ozler et al., 2015; Fuji et al., 2014), no head-to-head RCTs were found. The 25 RCTs include a total of 42,994 patients (22,882 in the NOACs group and 20,112 in the non NOAC group). The NOACs included apixaban (*n* = 4), rivaroxaban (*n* = 11), dabigatran (*n* = 7), edoxaban (*n* = 3), betrixaban (*n* = 1), and the non-NOAC group included placebo (*n* = 1), enoxaparin (*n* = 19), nadroparin (*n* = 1), aspirin (*n* = 3), and TB-402 (*n* = 1). We summarized the characteristics of the included RCTs (Table 1 and Supplementary Table S2). The mean age of participants was 64.06 years (ranged from 18 to 93), 59.92% of whom were women. Twenty trials were double-blinded (Eriksson et al., 2007; Eriksson et al., 2007; Lassen et al., 2007; Eriksson et al., 2008; Kakkar et al., 2008; Lassen et al., 2008; Ginsberg et al., 2009; Lassen et al., 2009; Turpie et al., 2009; Fuji et al., 2010; Lassen et al., 2010; Lassen et al., 2010; Eriksson et al., 2011; Verhamme et al., 2013; Fuji et al., 2014; Fuji et al., 2014; Mirdamadi et al., 2014; Fuji et al., 2015; Kim et al., 2016; Anderson et al., 2018), and two trials were open-label^34, 39^, one trial blinded to the NOAC group but unblinded to non-NOAC group (Turpie et al., 2009) and two trials were not clear (Zou et al., 2014; Jiang et al., 2018). Ten studies reported only on patients undergoing THA (Eriksson et al., 2007; Eriksson et al., 2008; Kakkar et al., 2008; Lassen et al., 2010; Eriksson et al., 2011; Verhamme et al., 2013; Fuji et al., 2014; Fuji et al., 2015; Kim et al., 2016; Jiang et al., 2018), and 13 studies reported only on patients undergoing TKA (Eriksson et al., 2007; Lassen et al., 2007; Lassen et al., 2008; Ginsberg et al., 2009; Lassen et al., 2009; Turpie et al., 2009; Turpie et al., 2009; Fuji et al., 2010; Lassen et al., 2010; Fuji et al., 2014; Jiang et al., 2014; Mirdamadi et al., 2014; Zou et al., 2014), 2 studies reported on both patients undergoing THA and patients undergoing TKA (Ozler et al., 2015; Anderson et al., 2018).

**TABLE 1:** Characteristics of the included 25 randomized clinical trials

Characteristics	Value
No. of participants	
Total	42,994
NOACs	22,882
Non NOAC	20,112
Age, mean (range), y	
Total	64.06 (18–93)
NOACs	64.2 (18–93)
Non NOAC	63.9 (18–93)
Female, No. (%)	
Total	59.92%
NOACs	60.22%
Non-NOAC	59.58%
Joint replacement population	
Both THA and TKA	2 studies
THA only	10 studies
TKA only	13 studies
Comparator	
Enoxaparin	19 studies
Aspirin	3 studies
Nadroparin	1 study
Placo	1 study
TB-402	1 study

NOAC, new oral anticoagulants; THA, total hip arthroplasty; TKA, total knee arthroplasty.

**FIGURE 2:**
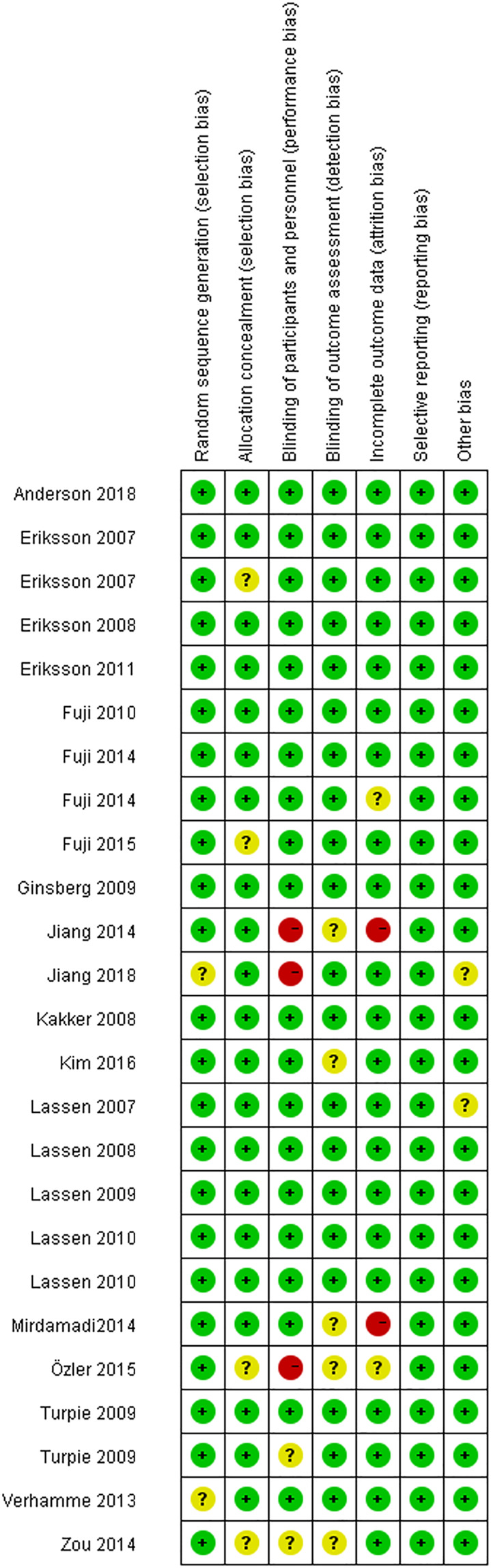
Risk of bias summary.

Figure 2 shows the results from the risk of bias assessment, most of the studies showed a lower risk of bias overall. Funnel plots of efficacy outcomes showed good symmetry, suggesting a small publication bias in VTE prophylaxis, while the funnel plot showed asymmetry regarding major bleeding, all bleeding, and clinically relevant nonmajor bleeding (Supplementary Figure S1).

### Outcomes of Direct Comparison Between NOACs and Non-NOAC

According to the results of direct comparison, use of NOACs show effective than non-NOAC in prevention of VTE [RR 0.68 (95% CI 0.55–0.84)], DVT [RR 0.65 (95% CI, 0.52–0.81)] and major VTE [RR = 0.52 (95% CI 0.35–0.76)]. Further subgroup analyses were conducted according to individual NOACs (rivaroxaban, apixaban, edoxaban, dabigatran, and betrixaban). Results show that rivaroxaban, apixaban, and edoxaban were obviously superior to non-NOAC in preventing VTE [RR 0.56 (95% CI 0.38–0.81); RR 0.61 (95% CI 0.39–0.95); RR 0.51 (95% CI 0.33–0.77)] and DVT [RR 0.65 (95% CI 0.52–0.81); RR 0.48 (95% CI 0.31–0.73); RR 0.56 (95% CI 0.35–0.89)]. Also, rivaroxaban showed excellent than non-NOAC in preventing major VTE [RR 0.30 (95% CI 0.16–0.57)]. Meanwhile, apixaban and betrixaban were more effective in preventing all bleeding [RR 0.88 (95% CI 0.79–0.99); RR 0.17 (95% CI 0.03–0.97)] than non-NOAC. There were no differences between NOACs and non-NOAC in all causes of death, PE, major bleeding, clinically relevant nonmajor bleeding, ischemic stroke, and myocardial infarction. The specific results are summarized in Table 2 and Supplementary Table S3.

**TABLE 2:** Forest plot results of any NOAC against non-NOAC in THA and TKA patients

Outcome	Comparison	RR (CI 95%)	Outcome	Comparison	RR (CI 95%)
VTE	NOACs vs. non-NOAC	**0.68 (0.55, 0.84)**	Major bleeding	NOACs vs. non-NOAC	1.14 (0.68, 1.91)
	Rivaroxaban vs. non-NOAC	**0.56 (0.38, 0.81)**		Rivaroxaban vs. non-NOAC	1.67 (0.48, 5.80)
	Apixaban vs. non-NOAC	**0.61 (0.39, 0.95)**		Apixaban vs. non-NOAC	0.82 (0.47, 1.41)
	Dabigatran vs. non-NOAC	0.95 (0.72, 1.27)		Dabigatran vs. non-NOAC	0.98 (0.67, 1.44)
	Edoxaban vs. non-NOAC	**0.51 (0.33, 0.77)**		Edoxaban vs. non-NOAC	1.03 (0.20, 5.42)
	Betrixaban vs. non-NOAC	1.51 (0.55, 4.12)		Betrixaban vs. non-NOAC	0.09 (0.00, 2.06)
All causes of death	NOACs vs. non-NOAC	0.95 (0.58, 1.56)	All bleeding	NOACs vs. non-NOAC	1.00 (0.91, 1.11)
	Rivaroxaban vs. non-NOAC	0.81 (0.40, 1.67)		Rivaroxaban vs. non-NOAC	1.07 (0.94, 1.21)
	Apixaban vs. non-NOAC	1.21 (0.48, 3.04)		Apixaban vs. non-NOAC	**0.88 (0.79, 0.99)**
	Dabigatran vs. non-NOAC	1.34 (0.33, 5.44)		Dabigatran vs. non-NOAC	1.19 (0.92, 1.54)
	—	—		Edoxaban vs. non-NOAC	1.21 (0.90, 1.62)
	—	—		Betrixaban vs. non-NOAC	**0.17 (0.03, 0.97)**
DVT	NOACs vs. non-NOAC	**0.65 (0.52, 0.81)**	CRNMB	NOACs vs. non-NOAC	1.04 (0.91, 1.18)
	Rivaroxaban vs. non-NOAC	**0.48 (0.31, 0.73)**		Rivaroxaban vs. non-NOAC	1.17 (0.92, 1.48)
	Apixaban vs. non-NOAC	**0.61 (0.40, 0.92)**		Apixaban vs. non-NOAC	0.83 (0.69, 1.01)
	Dabigatran vs. non-NOAC	0.91 (0.69, 1.21)		Dabigatran vs. non-NOAC	1.17 (0.94, 1.45)
	Edoxaban vs. non-NOAC	**0.56 (0.35, 0.89)**		Edoxaban vs. non-NOAC	1.27 (0.71, 2.27)
	Betrixaban vs. non-NOAC	1.38 (0.50, 3.80)		Betrixaban vs. non-NOAC	0.25 (0.04, 1.73)
PE	NOACs vs. non-NOAC	0.82 (0.54, 1.26)	Ischemic stroke	NOACs vs. non-NOAC	1.31 (0.61, 2.81)
	Rivaroxaban vs. non-NOAC	0.73 (0.25, 2.08)		Rivaroxaban vs. non-NOAC	1.84 (0.73, 4.65)
	Apixaban vs. non-NOAC	0.90 (0.23, 3.44)		Apixaban vs. non-NOAC	0.70 (0.14, 3.58)
	Dabigatran vs. non-NOAC	0.72 (0.33, 1.56)		—	—
	Edoxaban vs. non-NOAC	0.44 (0.10.2.04)		—	—
	Betrixaban vs. non-NOAC	1.19 (0.36, 3.90)		—	—
Major VTE	NOACs vs. non-NOAC	**0.52 (0.35, 0.76)**	Myocardial infarction	NOACs vs. non-NOAC	1.17 (0.68, 2.02)
	Rivaroxaban vs. non-NOAC	**0.30 (0.16, 0.57)**		Rivaroxaban vs. non-NOAC	1.06 (0.44, 2.52)
	Apixaban vs. non-NOAC	0.66 (0.31, 1.39)		Apixaban vs. non-NOAC	1.30 (0.51, 3.30)
	Dabigatran vs. non-NOAC	0.80 (0.53, 1.22)		Dabigatran vs. non-NOAC	0.99 (0.06, 15.85)

The cells contain the relative risk (RR), 95% confidence interval (CI) of the treatment comparison. **Bolded values** are statistically significant. VTE, venous thromboembolism; DVT, deep vein thrombosis; PE, pulmonary embolism; CRNMB, clinically relevant nonmajor bleeding.

### Outcomes of Network Comparison Among NOACs

**FIGURE 3:**
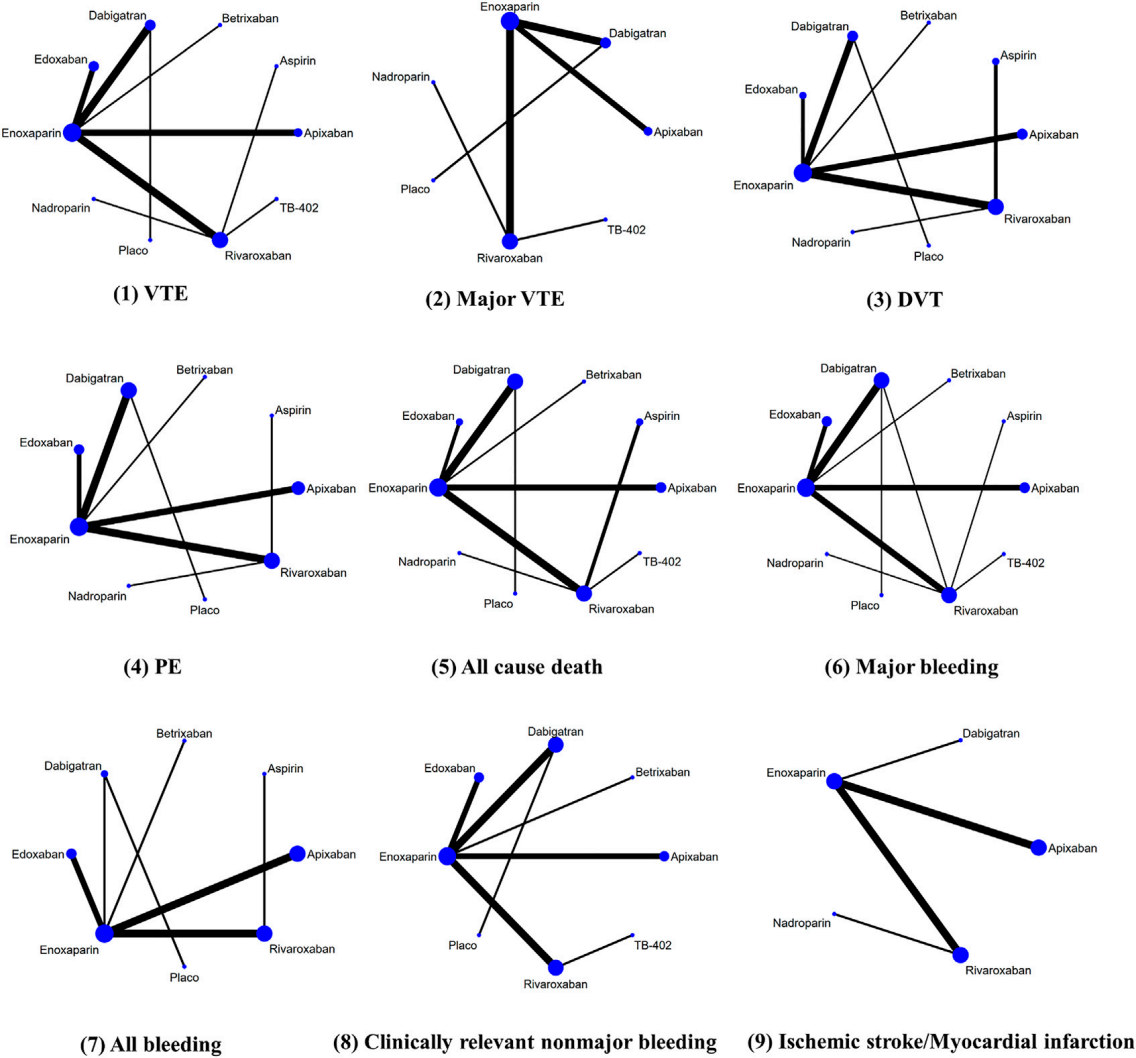
Network diagrams of primary and secondary outcomes in THA and TKA patients.

**FIGURE 4:**
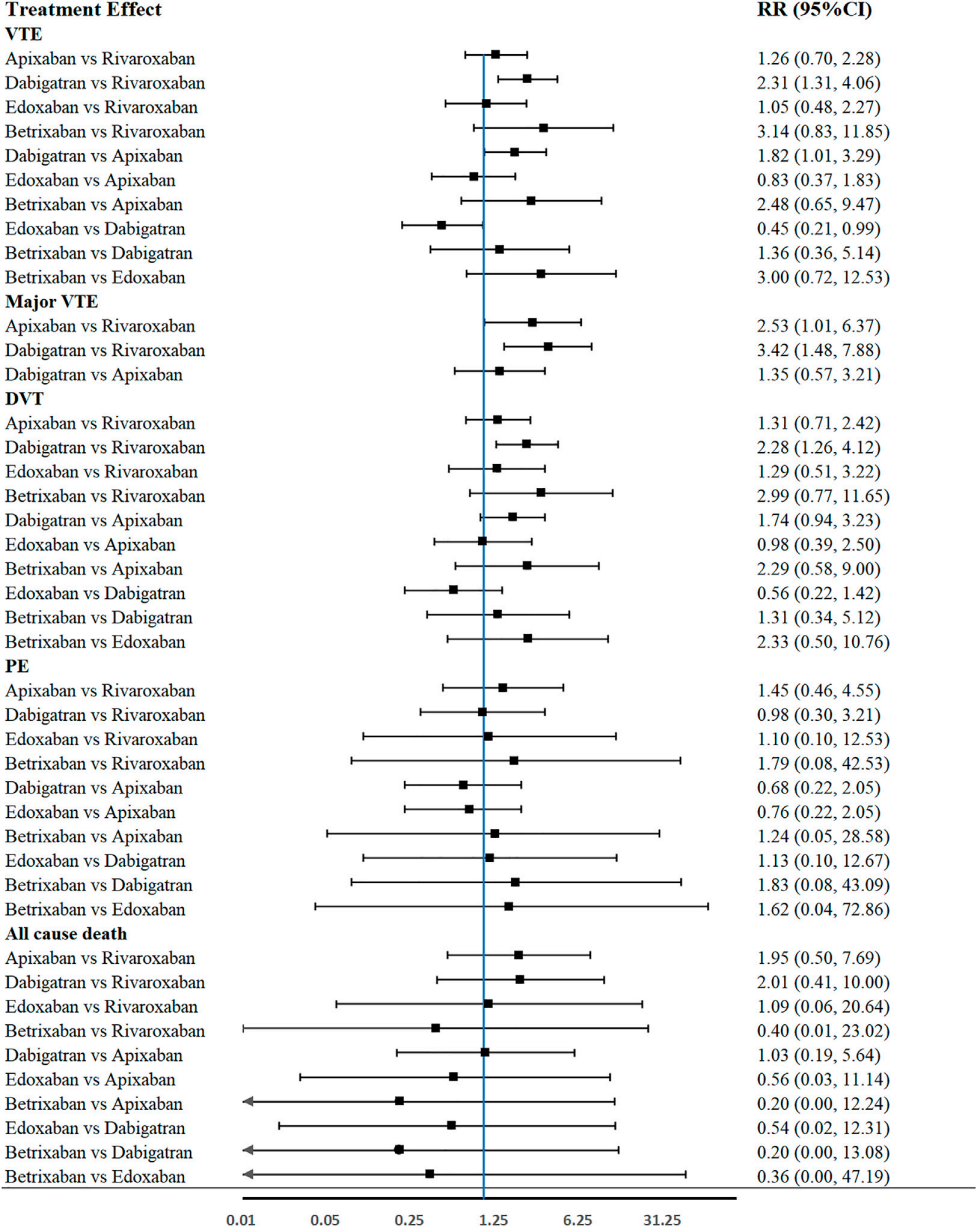
Forest plot of network meta-analysis of different NOACs for VTE, major VTE, DVT, PE, and all causes of death in THA and TKA patients.

**FIGURE 5:**
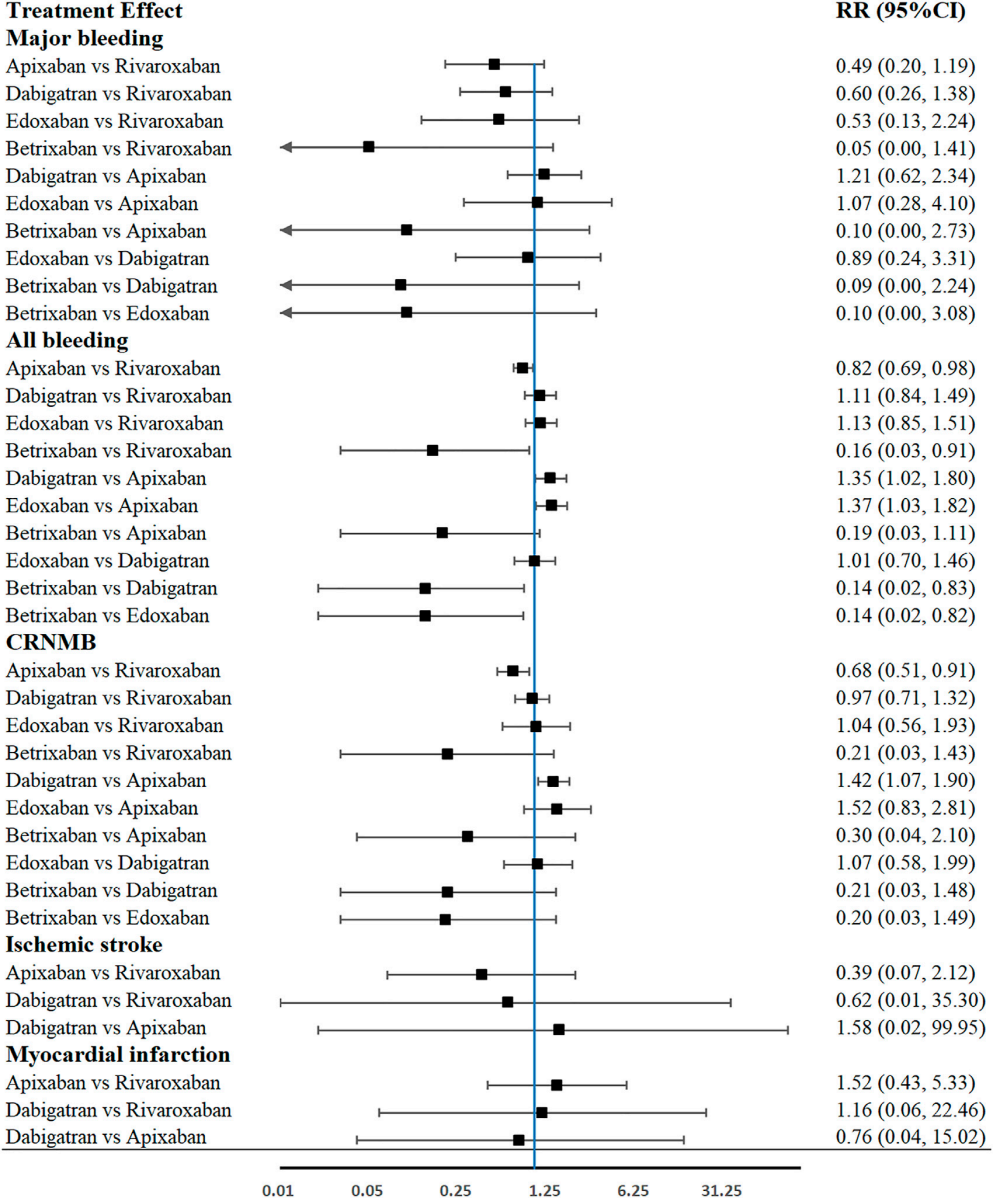
Forest plot of network meta-analysis of different NOACs for major bleeding, all bleeding, CRNMB, ischemic stroke, and myocardial infarction in THA and TKA patients. CRNMB, clinically relevant nonmajor bleeding.

Figure 3 provided the network diagrams, and non-NOAC was used as the common comparator across the studies. In overall analysis, rivaroxaban [RR 2.31 (95% CI 1.31–4.06)], apixaban [RR 1.82 (95% CI 1.01–3.29)], and edoxaban [RR 0.45 (95% CI 0.21–0.99)] were more effective than dabigatran in preventing VTE. Rivaroxaban [RR 2.28 (95% CI 1.26–4.12)] showed better than dabigatran in preventing DVT. Moreover, rivaroxaban also showed excellent than apixaban [RR 2.53 (95% CI 1.01–6.37)] and dabigatran [RR 3.42 (95% CI 1.48–7.88)] in major VTE prevention (Figure 4). With respect to safety outcome, apixaban and betrixaban were obviously superior to rivaroxaban, edoxaban, and dabigatran in all bleeding prevention, while no statistically significant differences were found between apixaban and betrixaban on this endpoint. Additionally, apixaban also show better effect than rivaroxaban [RR 0.68 (95%CI 0.51–0.91)] and dabigatran in clinically relevant nonmajor bleeding [RR 1.42 (95% CI 1.07–1.90)] (Figure 5).

### Relative Ranking of Anticoagulants

The clustered ranking plot depicted according to SUCRA values showed that rivaroxaban was the winner for the VTE (SUCRA = 79.6), DVT (SUCRA = 88.8), and major VTE (SUCRA = 89.9), while aspirin ranked first in all causes of death (SUCRA = 84.4) and PE (SUCRA = 65.5) prevention (Supplementary Table S4). Regarding safety outcome, nadroparin shows the most favorable position in major bleeding, followed by betrixaban and apixaban. Moreover, betrixaban is in the most favorable position in terms of all bleeding and clinically relevant nonmajor bleeding prevention, followed by apixaban, while nadroparin is superior to other anticoagulants in preventing ischemic stroke and myocardial infarction.

### Subgroup Analysis

Comparison of NOAC with conventional therapy was also performed in a subgroup classified by the type of joint surgery (THA vs. TKA) and the follow-up duration (<60 days vs. ≥ 60 days). In terms of the direct comparison (Supplementary Table S5), NOACs statistically significantly decreased the incidence of VTE, DVT, and major VTE compared with conventional therapy in patients with THA and patients with follow-up duration <60 days, consistent with overall analysis. Meanwhile, NOACs were associated with less VTE [RR 0.75 (95% CI 0.59–0.97)] and DVT [RR 0.73 (95% CI 0.57–0.93)] compared with non-NOAC in TKA patients but was not associated with decreased major VTE tendency. However, the NOACs showed no difference regarding efficacy and safety in studies follow-up duration ≥60 days.

In terms of the network comparison (Supplementary Table S6), the risk of major VTE of rivaroxaban was even lower than that of apixaban [RR 3.25 (95% CI, 1.10–9.66)] and dabigatran [RR 6.07 (95% CI, 2.63–14.05)] in THA patients. While in TKA patients, apixaban [RR 1.95 (95% CI, 1.18–3.21)] and edoxaban [RR 0.47 (95% CI, 0.22–0.98)] were more effective than dabigatran in preventing VTE, rivaroxaban [RR 1.93 (95% CI, 1.12–3.32)] was obviously superior to dabigatran in DVT prevention. Meanwhile, the risk of all bleeding of apixaban and betrixaban were even lower than rivaroxaban and edoxaban. Apixaban also shows excellent than rivaroxaban [RR 0.60 (95% CI 0.39–0.95)] in CRNMB prevention in TKA patients. Respect to patients with follow-up duration <60 days, betrixaban shows more effective than rivaroxaban [RR 0.15 (95% CI 0.39–0.95)] and edoxaban [RR 0.14 (95% CI 0.02–0.82)] in all bleeding prevention. Meanwhile, apixaban statistically significantly decreased the incidence of VTE, DVT, and CRNMB compared with dabigatran in patients with follow-up duration ≥60 days.

### Consistency and Sensitivity Analyses

Node-splitting analysis was applied to evaluate consistency by comparing the differences between direct and indirect evidence. After constructing the node-splitting model, we observed that there was no significant inconsistency in this study (Supplementary Table S7). Sensitivity analysis confirmed that the overall outcomes of DVT, PE, major VTE, and major bleeding failed to identify any individual trials as having influenced the results to a significant extent, confirming the robustness of the primacy findings (Supplementary Table S8).

## Discussion

Our systematic review and meta-analysis identified 25 RCTs that used NOACs for VTE prophylaxis in patients undergoing THA and TKA. We demonstrated that NOACs exhibited a higher efficacy (including VTE, DVT, and major VTE) and none of the individual NOACs increased the risk of bleeding when compared with non-NOAC, thereby validating the conclusion of no association between NOACs and increased risk of bleeding. Additionally, at the time of balancing efficacy and safety, the different anticoagulants also did tend to differ. Our network-pooled estimates of outcomes revealed that rivaroxaban may be the most favorable anticoagulant in terms of prevention of VTE, DVT, and major VTE, followed by aspirin and apixaban according to the SUCRA values, while nadroparin ranked first in terms of prevention of major bleeding. Meanwhile, aspirin had the highest-ranking position of SUCRA values in reducing all cause of death and PE. In addition, the top two interventions in terms of all bleeding and clinically relevant nonmajor bleeding prevention were betrixaban and apixaban.

Conventional therapy for patients undergoing THA and TKA surgery is perceived to be less clinically effective than NOACs interventions. In our study, NOACs significantly reduced the risk of VTE, DVT, and major VTE, compared to non-NOAC, with significant heterogeneity among included studies (I^2^ >50.0%, *p* < 0.05). Meanwhile, further subgroup analyses confirmed that rivaroxaban, apixaban, and edoxaban but not dabigatran and betrixaban, did confer a higher efficacy in our study. None of the individual NOACs increased the risk of bleeding, while apixaban and betrixaban were even associated with decreased risk of bleeding than non-NOAC. Although rivaroxaban, apixaban, and edoxaban are all considered to be effective for venous thromboembolism prophylaxis, rivaroxaban was significantly better than apixaban [RR 2.53 (95% CI 1.01–6.37)] and dabigatran [RR 3.42 (95% CI 1.48–7.88)] in major VTE prevention. The higher efficacy of rivaroxaban than apixaban may be attributed to the time of drug initiation: rivaroxaban was initiated 6–8 h after surgery in the RECORD trials, whereas apixaban was initiated 12–24 h after surgery in the ADVANCE trials, which may also contribute to apixaban decreased bleeding. Up to now, several systematic reviews and meta-analyses have been conducted to assess the effectiveness and safety risk of NOACs. Compared with conventional VTE prophylaxis, NOACs was also strongly supported to use in nonelective lower limb fracture surgery, such as after hip fracture (Waever et al., 2021). Similar to our study, Rezapour et al. confirmed that rivaroxaban was also shown to be more cost-effective than apixaban and dabigatran in the prevention of VTE after total knee and total hip replacement surgery (Rezapour et al., 2021). Al et al. showed that apixaban and rivaroxaban probably reduce the risk of recurrent hospitalization compared with vitamin K antagonists, additionally, dabigatran, apixaban, and rivaroxaban probably reduce non-major bleeding more than vitamin K antagonists (Al et al., 2019).

Interestingly, subgroup analysis confirmed that the clinical efficacy of the NOACs tended to be better in total hip replacement surgery than in total knee replacement surgery. Meanwhile, NOACs also associated with a higher efficacy in patients with follow-up duration <60 days than in patients with follow-up duration ≥60 days. Comparable efficacy and safety between NOACs therapy and conventional therapy was present in patients with follow-up duration ≥60 days in our study revealed that NOACs had a similar effectiveness and safety profile outcomes compared with other commonly used anticoagulants in the long term. However, larger sample RCT and real-world studies of high quality are needed to confirm the conclusion.

We aimed to evaluate the efficacy and safety of NOACs in THA and TKA patients. Given the absence of RCTs comparing different types of NOACs against each other, we conducted a network meta-analysis. This provided a comprehensive and comparative evaluation of all available treatment options in a coherent and methodologically robust way across efficacy and safety outcomes. We combined both direct and indirect evidence, thus increasing the statistical power and confidence in the results. Although several systematic reviews and meta-analysis have been conducted to assess the effectiveness and safety of NOACs after THA and TKA, the outcome data indicated RCTs they included were limited. The new anticoagulant was usually analyzed as individual NOACs or a single integrated group compared with enoxaparin in previous meta-analysis (Venker et al., 2017a; Kapoor et al., 2017; Yu et al., 2018). Several network meta-analysis report indirect comparisons provide head-to-head comparisons of new oral anticoagulants, such as apixaban, rivaroxaban, and dabigatran with or without edoxaban (Gomez-Outes et al., 2012; Hur et al., 2017; Feng et al., 2021). However, those studies did not provide comparison with aspirin and nadroparin, and our study may change the interpretation of existing data.

Considerable debate surrounds the use of aspirin in THA and TKA patients for venous thromboembolism prophylaxis. The inclusion of a recent large RCT (Anderson et al., 2018) in this meta-analysis was important given that it was large sample, high quality, and represented one of only three studies^30, 34, 35^ that appraised the newer oral anticoagulants with aspirin. Moreover, this RCT has not been considered in any previous meta-analysis to date on the same topic and thus could change the interpretation of existing data. A recent meta-analysis is done for clinical effectiveness and safety of aspirin suggests that it did not differ statistically significantly from other anticoagulants used for VTE prophylaxis after THA and TKA (Matharu et al., 2020). Our network-pooled estimates suggest that aspirin could in fact be effective in VTE prevention, where it showed best efficacy in all causes of death and PE prevention. However, aspirin could be suboptimal for bleeding problems prophylaxis, as it ranks poorly than other anticoagulants in terms of safety outcomes.

Notably, the DVT outcome data in our analysis included both symptomatic and asymptomatic events, rather than solely symptomatic events that are often deemed as more clinically relevant in other studies. In THA and TKA surgery, differentiation between symptomatic and asymptomatic deep vein thrombosis can be problematic and so under-diagnosis of deep vein thrombosis is a possibility (Lewis et al., 2019a; Lewis et al., 2019b). Also, symptomatic DVT are commonly rare, which means the number of DVT events included in our analysis would be small, resulting in network analysis with sparse data and unstable results. This potential source of heterogeneity should be considered. Also, the primary endpoint in the meta-analysis by Gómez-Outes et al. was symptomatic VTE (Gomez-Outes et al., 2012), while all VTEs were included as primary endpoint in our analysis. They concluded that rivaroxaban halved the risk of symptomatic VTEs (RR = 0.48, 95% CI 0.31–0.75), and the RR we calculated for rivaroxaban was similar (0.56, 95% CI 0.46–0.66).

To our knowledge, our study presents the most up to date and comprehensive systematic review and network meta-analysis done to compare the efficacy and safety of NOACs (including apixaban, rivaroxaban, edoxaban, dabigatran, and betrixaban) with non-NOAC for VTE prophylaxis after THA and TKA, and we also ranked the different treatments according to the SUCRA values. Sensitivity analyses conducted for the primary analysis suggested the robustness of our study. Previous studies confirmed that a higher efficacy of new anticoagulants was generally associated with a higher bleeding tendency (Gomez-Outes et al., 2012; Venker et al., 2017b; Hur et al., 2017; Yu et al., 2018). While our study confirmed that new anticoagulants exhibited a higher efficacy without a higher bleeding tendency. Variations in definition of hemorrhage endpoints may explain the differences between our analysis and prior studies. The hemorrhage endpoint in the meta-analysis by Gomez-Outes et al. (2012) and Venker et al. (2017a) was major/clinically relevant nonmajor bleeding and major/clinically relevant bleeding, respectively, while all bleeding, major bleeding, and clinically relevant nonmajor bleeding were included as safety endpoints in our analysis. Moreover, an important difference between our study and prior meta-analysis is that we included betrixaban, aspirin, and nadroparin. Some limitations of this study need to be acknowledged. First, the RCTs had incongruent drug administration and follow-up durations, although the risk of VTE and hemorrhage after surgery persists for months. Common to most pooled analyses is the lack of individual patient data. Thus, we were compelled to select summary RR for analysis by measuring only the number of events and taking no account of when they occurred. Second, the inconsistency in outcome definitions in the included RCTs, particularly for the hemorrhage outcome, is an inherent limitation in the network meta-analysis area and future trials should use standardized definitions. Third, findings for betrixaban and nadroparin were based on data reported by few studies, and some of the estimates were imprecise. In the EXPERT trial, the betrixaban dosage was blinded, but enoxaparin was not. Therefore, caution is needed when interpreting these results. Hence, the present results must be interpreted with caution in light of the above-mentioned limitations.

## Conclusion

In conclusion, the present data indicate that NOACs show better efficacy than non-NOAC in venous thromboembolism prevention. None of the individual NOACs increased the risk of bleeding when compared with non-NOAC, while apixaban and betrixaban were even associated with decreased risk of bleeding. These results provide a comprehensive assessment of relative efficacy and their associated uncertainty, which could be used to balance the benefit-risk of new oral anticoagulants in arthroplasty.

## Data Availability

The original contributions presented in the study are included in the article/Supplementary Material, Further inquiries can be directed to the corresponding authors.
