# Supplementary material for: New Oral Anticoagulants for Venous Thromboembolism Prophylaxis in Total Hip and Knee Arthroplasty: A Systematic Review and Network Meta-Analysis
Source: Front Pharmacol. 2022 Jan 17;12:775126. doi: 10.3389/fphar.2021.775126 (PMC8801787; doi:10.3389/fphar.2021.775126)
Supplement: Supplementary file 1 [file DataSheet1.docx]

**SUPPLEMENTAL MATERIAL**

**Supplementary Table 1** Search strategy -------------------------------------------------------------------------------------- 2

**Supplementary Table 2** Summary characteristics of the included 25 randomized clinical trials---------------------- 4

**Supplementary Figure 1** Funnel plot of (A)VTE, (B) Major VTE, (C) DVT, (D) PE, (E) Major VTE, (F) Major bleeding, (G) All bleeding, and (H) Clinically relevant nonmajor bleeding in total hip arthroplasty and total knee arthroplasty patients ---------------------------------------------------------------------------------------------------------------9

**Supplementary Table 3** Relative risk outputs of the direct comparison in THA and TKA patients ----------------------------------------------------------------------------------------------------------------------------------------------------------- 10

**Supplementary Table 4** SUCRA ranking of different NOACs and conventional anticoagulants --------------------------------------------------------------------------------------------------------------------------------------------------------------15

**Supplementary Table 5** Forest plot results of direct analysis for different outcomes in subgroups ---------------------------------------------------------------------------------------------------------------------------------------------------------- 17

**Supplementary Table 6** Network meta-analysis of the subgroups ------------------------------------------------------ 19

**Supplementary Table 7** Results of node-splitting models------------------------------------------------------------------24

**Supplementary Table 8** Sensitivity analyses -------------------------------------------------------------------------------- 26

**Supplementary Table 1** Search strategy

1. **Medline**

| #1 | "non vitamin k antagonist oral anticoagulant"[Title/Abstract] OR "non vitamin k antagonist oral anticoagulants"[Title/Abstract] OR "novel oral anticoagulant"[Title/Abstract] OR "novel oral anticoagulants"[Title/Abstract] OR "new oral anticoagulant"[Title/Abstract] OR "new oral anticoagulants"[Title/Abstract] OR "NOAC"[Title/Abstract] OR "NOACs"[Title/Abstract] | 4598 |
| --- | --- | --- |
| 2 | "Rivaroxaban"[Title/Abstract] OR "Xarelto"[Title/Abstract] OR "bay 59-7939"[Title/Abstract] OR "bay 59 7939"[Title/Abstract] OR "BAY 597939"[Title/Abstract] | 5481 |
| #3 | "Apixaban"[Title/Abstract] OR "Eliquis"[Title/Abstract] OR "bms 562247"[Title/Abstract] OR "BMS562247"[Title/Abstract] OR "bms-562247"[Title/Abstract] | 3501 |
| #4 | "Betrixaban"[Title/Abstract] OR "Bevyxxa"[Title/Abstract] OR "PRT054021"[Title/Abstract] | 176 |
| #5 | "edoxaban"[Title/Abstract] OR "Lixiana"[Title/Abstract] OR "Savaysa"[Title/Abstract] OR "DU-176"[Title/Abstract] OR "DU-176b"[Title/Abstract] | 1498 |
| #6 | "dabigatran"[Title/Abstract] OR "Pradaxa"[Title/Abstract] OR "bibr 1048"[Title/Abstract] | 4995 |
| #7 | #1 OR #2 OR #3 OR #3 OR #4 OR #5 OR #6 | 11888 |
| #8 | "hip"[All Fields] OR "knee"[All Fields] OR "orthopedic operation"[All Fields] OR "orthopedic surgery"[All Fields] | 348603 |
| #9 | #7 AND #8 | 824 |

1. **Embase**

| #1 | 'non vitamin k antagonist oral anticoagulant':ab,ti OR 'non vitamin k antagonist oral anticoagulants':ab,ti OR 'novel oral anticoagulant':ab,ti OR 'novel oral anticoagulants':ab,ti OR 'new oral anticoagulant':ab,ti OR 'new oral anticoagulants':ab,ti OR noac:ab,ti OR noacs:ab,ti | 8323 |
| --- | --- | --- |
| #2 | rivaroxaban:ab,ti OR xarelto:ab,ti OR 'bay 59-7939':ab,ti OR 'bay 59 7939':ab,ti OR 'bay 597939':ab,ti | 10888 |
| #3 | apixaban:ab,ti OR eliquis:ab,ti OR bms562247:ab,ti OR 'bms 562247':ab,ti | 7063 |
| #4 | betrixaban:ab,ti OR bevyxxa:ab,ti OR prt054021:ab,ti | 273 |
| #5 | edoxaban:ab,ti OR lixiana:ab,ti OR savaysa:ab,ti OR 'du 176':ab,ti OR 'du 176b':ab,ti | 2456 |
| #6 | dabigatran:ab,ti OR pradaxa:ab,ti OR 'bibr 1048':ab,ti | 9632 |
| #7 | #1 OR #2 OR #3 OR #3 OR #4 OR #5 OR #6 | 22667 |
| #8 | hip OR knee OR 'rthopedic operation' OR 'orthopedic surgery' | 513090 |
| #9 | #7 AND #8 | 1815 |

1. **Cochrane-CENTRAL**

| #1 | non vitamin k antagonist oral anticoagulant OR non vitamin k antagonist oral anticoagulants OR novel oral anticoagulant OR novel oral anticoagulants OR new oral anticoagulant in Title Abstract Keyword | 998 |
| --- | --- | --- |
| #2 | new oral anticoagulants OR noac OR noacs OR rivaroxaban OR xarelto in Title Abstract Keyword | 2190 |
| #3 | bay 59 7939 OR bay 597939 OR apixaban OR eliquis OR bms562247 | 973 |
| #4 | bms 562247 OR betrixaban OR bevyxxa OR prt054021 OR edoxaban | 659 |
| #5 | lixiana OR savaysa OR du 176 OR du 176b OR dabigatran | 1083 |
| #6 | pradaxa OR bibr 1048 | 88 |
| #7 | #1 OR #2 OR #3 OR #3 OR #4 OR #5 OR #6 | 3994 |
| #8 | hip OR knee OR rthopedic operation OR orthopedic surgery | 58727 |
| #9 | #7 AND #8 | 427 |

**Supplementary Table 2** Summary characteristics of the included 25 randomized clinical trials

| **Study** | **Patients** | **Sample size** | **Mean age (y)** | **Female (%)** | **Countries** | **Intervention** | **Comparison** | **Follow-up** | **Effectiveness** | **Safety** |
| --- | --- | --- | --- | --- | --- | --- | --- | --- | --- | --- |
| Eriksson 2008 | THA | 4433 | 63.2 | 55.5 | Multicenter  27 countries in worldwide | Intervention(n=2209): Rivaroxaban 10 mg, qd., p.o.. administered until day 35. | Comparison (n=1599):  Enoxaparin 40mg, qd, s.c.. administered until day 35. | 30-35days after last dose of study drug. | Favors rivaroxaban | No significant difference |
| Kakker  2008 | THA | 2509 | 61.5 | 53.6 | Multicenter  23 centres  In 21 countries | Intervention(n=1252): Rivaroxaban 10 mg, qd., p.o.. administered until day 31-39. | Comparison (n=1257):  Enoxaparin 40mg, qd, s.c.. administered until day 10-14 days. | 30-35days after last dose of study drug. | Favors rivaroxaban | No significant difference |
| Lassen  2008 | TKA | 2531 | 67.6 | 68.3 | Multicenter  147 centers in 19 countries | Intervention(n=1254): Rivaroxaban 10 mg, qd., p.o.. administered until day 10-14. | Comparison (n=1277):  Enoxaparin 40mg, qd, s.c.. administered until day 10-14 days. | 30-35days after last dose of study drug. | Favors rivaroxaban | No significant difference |
| Turpie  2009 | TKA | 3034 | 64.5 | 65 | 131 centers in 12 countries | Intervention(n=1526): Rivaroxaban 10 mg, q.d., p.o.. administered until day 11-15. | Comparison (n=1508):  Enoxaparin 30mg, q12h, s.c.. administered until day 10-14 days. | 30-35 days after last dose of study drug. | Favors rivaroxaban | No significant difference |
| Lassen  2007 | TKA | 462 | 67.3 | 65.0 | Multicenter  97 centres in Argentina, Australia,  Canada, Mexico, Denmark, Israel, Poland, and the USA. | Intervention(n=310): Apixaban 2.5mg bid or apixaban 5mg qd., p.o.. administered until day 12. | Comparison (n=152):  Enoxaparin 30mg, q12h, s.c.. administered until day 12. | 30 days after last dose of study drug. | Favors apixaban | No significant difference |
| Lassen  2009 | TKA | 3195 | 65.8 | 62.1 | Multicenter  129 sites in 14 countries | Intervention(n=1599): Apixaban 2.5mg, bid., p.o.. administered until day 10-14. | Comparison (n=1596):  Enoxaparin 30mg, q12h, s.c.. administered until day 10-14. | 60 days after last dose of study drug. | No significant difference | Apixaban associated with lower rates of clinically relevant bleeding. |
| Lassen  2010 | TKA | 3057 | 67 | 72.5 | Multicenter  125 sites in 27 countries. | Intervention(n=1528): Apixaban 2.5mg, bid., p.o.. administered until day 10-14. | Comparison (n=1529):  Enoxaparin 40mg, qd, s.c.. administered until day 10-14. | 60 days after last dose of study drug. | Favors apixaban | No significant difference |
| Lassen  2010 | THA | 5407 | 60.8 | 53.3 | Multicenter  160 sites in 21 countries | Intervention(n=2708): Apixaban 2.5mg, bid., p.o.. administered until day 32-38. | Comparison (n=2699):  Enoxaparin 40mg, qd, s.c.. administered until day 32-38. | 60 days after last dose of study drug. | Favors apixaban | No significant difference |
| Eriksson 2007 | THA | 3463 | 64 | 56.3 | Multicenter  115 centres in  Europe, Australia, and South Africa | Intervention(n=2309): Dabigatran150mg or 220mg, qd., p.o.. administered until day 28-35. | Comparison (n=1154):  Enoxaparin 40mg, qd, s.c.. administered until day 28-35. | 60 days after last dose of study drug. | No significant difference | No significant difference |
| Eriksson 2011 | THA | 2013 | 62 | 51.8 | Multicenter  108 centres in 19 countries | Intervention(n=1010): Dabigatran 220mg, qd., p.o.. administered until day 28-35. | Comparison (n=1003):  Enoxaparin 40mg, qd, s.c.. administered until day 28-35. | 3 months after surgery | Favors dabigatran | No significant difference |
| Eriksson 2007 | TKA | 2076 | 67.7 | 66 | Multicenter  105 centers in Europe, Australia, and South Africa | Intervention(n=1382): Dabigatran150mg or 220mg, qd., p.o.. administered until day 6-10. | Comparison (n=679):  Enoxaparin 40mg, qd, s.c.. administered until day 6-10. | 3 months after surgery | No significant difference | No significant difference |
| Ginsberg 2009 | TKA | 2596 | 66.1 | 57.7 | Multicenter  58 centers in the United States, 30 in Canada, 8 in  Mexico, and 1 in the United Kingdom. | Intervention(n=1728): Dabigatran150mg or 220mg, qd., p.o.. administered until day 12-15. | Comparison (n=868):  Enoxaparin 30mg, q12h, s.c.. administered until day 12-15. | 3 months after surgery | Favors enoxaparin | No significant difference |
| Fuji  2010 | TKA | 379 | 71.6 | 83.8 | Multicenter  38 center in Japan | Intervention(n=255): Dabigatran150mg or 220mg, qd., p.o.. administered until day 11-14. | Comparison (n=124):  Placebo, qd., p.o.. administered until day 12-15. | 7-10 days after last dose of study drug. | Favors dabigatran | No significant difference |
| Turpie  2009 | TKA | 214 | 64.6 | 60.3 | Multicenter | Intervention(n=171):  Betrixaban 15mg or 40mg, bid., p.o.. administered until day 10-14. | Comparison (n=43):  Enoxaparin 30mg, q12h, s.c.. administered until day 10-14. | 6 weeks after surgery | No significant difference | No significant difference |
| Anderson 2018 | TKA+THA | 3424 | 62.8 | 52.2 | Multicenter  15 centers in Canada. | Intervention(n=1717): Rivaroxaban 10 mg, qd., p.o.. administered until day 14 (TKA ) and day35 (THA). | Comparison (n=1707):  Rivaroxaban 10 mg, qd., p.o.. administered until day 5, then aspirin 81 mg, qd, p.o.. administered until day 14 (TKA ) and day35 (THA). | 3 months after surgery | No significant difference | No significant difference |
| Fuji  2014 | TKA | 594 | 72.4 | 79.8 | Multicenter in Japan and Taiwan | Intervention(n=299):  Edoxaban 30mg qd., p.o.. administered until day 11-14. | Comparison (n=295):  Enoxaparin 2000 IU, q12h, s.c.. administered until day 11-14. | 25-35 days after last dose of study drug. | Favors edoxaban | No significant difference |
| Fuji  2015 | THA | 610 | 62.8 | 86 | Multicenter in Japan | Intervention(n=307):  Edoxaban 30mg qd., p.o.. administered until day 11-14. | Comparison (n=303):  Enoxaparin 2000 IU, q12h, s.c.. administered until day 11-14. | 25-35 days after last dose of study drug. | Favors edoxaban | No significant difference |
| Kim  2016 | THA | 701 | 56 | 52 | Single center in  Korea | Intervention(n=350): Rivaroxaban 10 mg, qd., p.o.. | Comparison (n=351):  Aspirin 81 mg, qd, p.o.. administered until day 7-12. | 6 weeks after surgery | No significant difference | / |
| Jiang  2014 | TKA | 120 | 64.5 | 92.5 | Single center in  China | Intervention(n=60):  LMWH 5000 U s.c.. administered until days 1-5, then rivaroxaban 10 mg, qd., p.o.. until day 14. | Comparison (n=60):  Aspirin 100 mg, qd, p.o.. administered until day 1-14. | 6 weeks after surgery | No significant difference | / |
| Zou  2014 | TKA | 214 | 63.1 | 71.8 | Single center in  China | Intervention(n=102): Rivaroxaban 10 mg, qd., p.o.. administered until day 14. | Comparison (n=110):  Aspirin 100 mg, qd, p.o.. administered until day 14. | 4 weeks | Favors rivaroxaban | / |
| Verhamme 2013 | THA | 622 | 62 | 55 | Multicenter | Intervention(n=207): Rivaroxaban 10 mg, qd., p.o.. administered until day 35. | Comparison (n=415):  TB-402 25mg or 50mg, qd, i.v.. administered until day 35. | 3 months after surgery | No significant difference | Favors rivaroxaban |
| Jiang  2018 | THA | 831 | 61.03 | 53 | Single center in  China | Intervention(n=277): Rivaroxaban 10 mg, qd., p.o.. administered until day 35. | Comparison (n=554):  Nadroparin 2000 IU, q12h, s.c.. administered until day 35. | 40 days after surgery | No significant difference | Favors nadroparin |
| Mirdamadi  2014 | TKA | 90 | 70.2 | 64.4 | Single center in  Iran | Intervention(n=45): Dabigatran 150 mg p.o..started 4 h after surgery and continued with 225 mg daily to 15 days. | Comparison (n=45):  Enoxaparin 40mg, q12h,s.c.. administered until day 15. | 3 months after surgery | No significant difference | No significant difference |
| Özler  2015 | TKA+THA | 180 | 66.7 | 61.1 | Single center in  Turkey | Intervention1 (n=60):  2×0.3-mL enoxaparin during the hospital stay and 10 mg rivaroxaban during the outpatient period for 10 days (TKA) and 30 days (THA).  Intervention2 (n=60):  2×0.3-mL enoxaparin during the hospital stay and 220 mg dabigatran during the outpatient period for 10 days (TKA) and 30 days (THA). | Intervention(n=60):  2×0.3-mL enoxaparin during the hospitalization period, and 1×0.4-mL enoxaparin during the outpatient period for 10 days (TKA) and 30 days (THA). | Comparison (n=45):  Enoxaparin 40mg, q12h,s.c.. administered until day 15. | No significant difference | No significant difference |
| Fuji  2014 | THA | 241 |  |  | Multicenter in Japan and Taiwan | Intervention1 (n=174):  Edoxaban 15 or 30mg qd., p.o.. administered until day 11-14. | Intervention1 (n=87):  Enoxaparin 2000 IU, q12h, s.c.. administered until day 11-14. | 25-35 days | No significant difference | No significant difference |

**
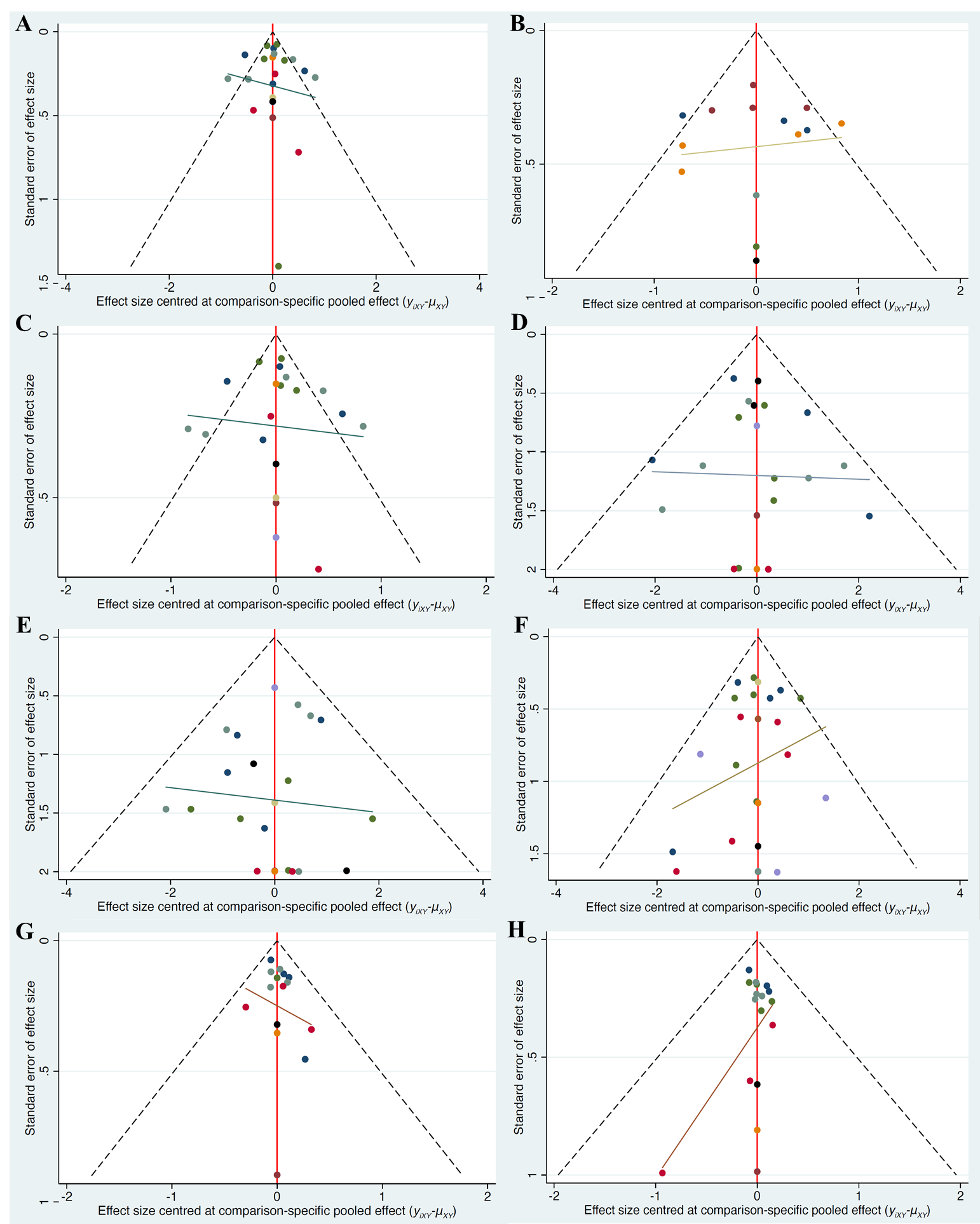
Supplementary Figure 1** Funnel plot of (A)VTE, (B) Major VTE, (C) DVT, (D) PE, (E) All cause death, (F) Major bleeding, (G) All bleeding, and (H) Clinically relevant nonmajor bleeding in total hip arthroplasty and total knee arthroplasty patients. Funnel plot was generated if the number of included studies ≥ 10.

**Supplementary Table 3** Relative risk outputs of the direct comparison in THA and TKA patients

1. **VTE**

| Study | t1 | t2 | RR (95% CI) | ^a^Weigh (%) |
| --- | --- | --- | --- | --- |
| 1 | Rivaroxaban | Enoxaparin | 0.30 (0.17, 0.52) | 4.56 |
| 2 | Rivaroxaban | Enoxaparin | 0.20 (0.12, 0.35) | 4.59 |
| 3 | Rivaroxaban | Enoxaparin | 0.49 (0.38, 0.63) | 5.99 |
| 4 | Rivaroxaban | Enoxaparin | 0.71 (0.51, 0.98) | 5.71 |
| 5 | apixaban | Enoxaparin | 0.61 (0.33, 1.11) | 4.29 |
| 6 | apixaban | Enoxaparin | 1.04 (0.79, 1.36) | 5.94 |
| 7 | apixaban | Enoxaparin | 0.60 (0.49, 0.72) | 6.23 |
| 8 | apixaban | Enoxaparin | 0.33 (0.21, 0.52) | 5.05 |
| 9 | dabigatran | Enoxaparin | 1.32 (0.96, 1.81) | 5.73 |
| 10 | dabigatran | Enoxaparin | 0.89 (0.64, 1.25) | 5.66 |
| 11 | dabigatran | Enoxaparin | 1.03 (0.89, 1.19) | 6.36 |
| 12 | dabigatran | Enoxaparin | 1.25 (1.06, 1.47) | 6.32 |
| 13 | dabigatran | placo | 0.49 (0.36, 0.66) | 5.82 |
| 14 | betrixaban | Enoxaparin | 1.51 (0.55, 4.12) | 2.69 |
| 15 | Rivaroxaban | Aspirin | 1.09 (0.48, 2.45) | 3.37 |
| 16 | edoxaban | Enoxaparin | 0.53 (0.32, 0.87) | 4.87 |
| 17 | edoxaban | Enoxaparin | 0.35 (0.14, 0.87) | 2.98 |
| 18 | Rivaroxaban | Enoxaparin | 1.09 (0.64, 1.85) | 4.66 |
| 21 | Rivaroxaban | TB-402 | 0.90 (0.42, 1.95) | 3.56 |
| 22 | Rivaroxaban | Nadroparin | 0.52 (0.23, 1.18) | 3.35 |
| 23 | dabigatran | Enoxaparin | 1.00 (0.07, 15.50) | 0.56 |
| 25 | edoxaban | Enoxaparin | 0.83 (0.20, 3.41) | 1.71 |
| Overall  (I^2^=86.0%, p=0.000) | NOACs | non-NOAC | **0.68 (0.55, 0.84)** | 100.00 |

^a^ Weights are from random effects analysis

1. **All cause death**

| Study | t1 | t2 | RR (95% CI) | ^a^Weigh (%) |
| --- | --- | --- | --- | --- |
| 1 | Rivaroxaban | Enoxaparin | 1.26 (0.34, 4.68) | 10.97 |
| 2 | Rivaroxaban | Enoxaparin | 0.25 (0.05, 1.18) | 8.39 |
| 3 | Rivaroxaban | Enoxaparin | 0.08 (0.00, 1.39) | 2.74 |
| 4 | Rivaroxaban | Enoxaparin | 0.99 (0.32, 3.06) | 13.83 |
| 5 | apixaban | Enoxaparin | 1.48 (0.06, 36.02) | 2.24 |
| 6 | apixaban | Enoxaparin | 0.50 (0.13, 1.99) | 10.09 |
| 7 | apixaban | Enoxaparin | 3.00 (0.31, 28.83) | 4.29 |
| 8 | apixaban | Enoxaparin | 2.49 (0.48, 12.83) | 7.61 |
| 9 | dabigatran | Enoxaparin | 6.50 (0.37, 115.28) | 2.74 |
| 10 | dabigatran | Enoxaparin | 0.20 (0.01, 4.13) | 2.47 |
| 11 | dabigatran | Enoxaparin | 1.00 (0.09, 11.06) | 3.85 |
| 12 | dabigatran | Enoxaparin | 2.51 (0.12, 52.29) | 2.47 |
| 15 | Rivaroxaban | Aspirin | 5.97 (0.72, 49.50) | 4.84 |
| 21 | Rivaroxaban | TB-402 | 2.01 (0.13, 31.89) | 2.95 |
| 22 | Rivaroxaban | Nadroparin | 0.67 (0.29, 1.55) | 20.54 |
| Overall  (I^2^=13.1%, p=0.307) | NOACs | non-NOAC | 0.95 (0.58, 1.56) | 100.00 |

^a^ Weights are from random effects analysis

1. **DVT**

| Study | t1 | t2 | RR (95% CI) | ^a^Weigh (%) |
| --- | --- | --- | --- | --- |
| 1 | Rivaroxaban | Enoxaparin | 0.23 (0.13, 0.43) | 4.62 |
| 2 | Rivaroxaban | Enoxaparin | 0.20 (0.11, 0.35) | 4.79 |
| 3 | Rivaroxaban | Enoxaparin | 0.50 (0.39, 0.65) | 6.37 |
| 4 | Rivaroxaban | Enoxaparin | 0.72 (0.51, 1.00) | 5.99 |
| 5 | apixaban | Enoxaparin | 0.69 (0.36, 1.29) | 4.45 |
| 6 | apixaban | Enoxaparin | 0.97 (0.73, 1.28) | 6.26 |
| 7 | apixaban | Enoxaparin | 0.59 (0.48, 0.71) | 6.61 |
| 8 | apixaban | Enoxaparin | 0.32 (0.20, 0.52) | 5.27 |
| 9 | dabigatran | Enoxaparin | 1.04 (0.76, 1.41) | 6.14 |
| 10 | dabigatran | Enoxaparin | 0.89 (0.64, 1.25) | 6 |
| 11 | dabigatran | Enoxaparin | 1.03 (0.89, 1.19) | 6.76 |
| 12 | dabigatran | Enoxaparin | 1.27 (1.08, 1.50) | 6.7 |
| 13 | dabigatran | placo | 0.49 (0.36, 0.66) | 6.19 |
| 14 | betrixaban | Enoxaparin | 1.38 (0.50, 3.80) | 2.86 |
| 16 | edoxaban | Enoxaparin | 0.53 (0.32, 0.87) | 5.2 |
| 18 | Rivaroxaban | Enoxaparin | 1.05 (0.60, 1.82) | 4.88 |
| 19 | Rivaroxaban | Aspirin | 1.10 (0.51, 2.40) | 3.77 |
| 20 | Rivaroxaban | LMWH | 0.18 (0.06, 0.59) | 2.33 |
| 22 | Rivaroxaban | Nadroparin | 0.56 (0.21, 1.48) | 2.97 |
| 25 | edoxaban | Enoxaparin | 0.83 (0.20, 3.41) | 1.84 |
| Overall  (I^2^=86.9%, p=0.000) | NOACs | non-NOAC | **0.65 (0.52, 0.81)** | 100.00 |

^a^ Weights are from random effects analysis

1. **PE**

| Study | t1 | t2 | RR (95% CI) | ^a^Weigh (%) |
| --- | --- | --- | --- | --- |
| 1 | Rivaroxaban | Enoxaparin | 4.03 (0.45, 36.00) | 3.50 |
| 2 | Rivaroxaban | Enoxaparin | 0.25 (0.03, 2.24) | 3.50 |
| 3 | Rivaroxaban | Enoxaparin | 0.11 (0.01, 2.10) | 2.03 |
| 4 | Rivaroxaban | Enoxaparin | 0.62 (0.20, 1.88) | 11.27 |
| 5 | apixaban | Enoxaparin | 0.10 (0.01, 2.04) | 1.89 |
| 6 | apixaban | Enoxaparin | 1.41 (0.68, 2.95) | 20.06 |
| 7 | apixaban | Enoxaparin | 7.01 (0.86, 56.86) | 3.80 |
| 8 | apixaban | Enoxaparin | 0.33 (0.09, 1.23) | 8.75 |
| 9 | dabigatran | Enoxaparin | 1.00 (0.25, 3.99) | 7.93 |
| 10 | dabigatran | Enoxaparin | 0.50 (0.05, 5.47) | 2.95 |
| 11 | dabigatran | Enoxaparin | 0.50 (0.03, 8.02) | 2.25 |
| 12 | dabigatran | Enoxaparin | 0.60 (0.18, 1.97) | 10.25 |
| 14 | betrixaban | Enoxaparin | 1.28 (0.06, 26.16) | 1.91 |
| 15 | Rivaroxaban | Aspirin | 1.19 (0.37, 3.90) | 10.24 |
| 18 | Rivaroxaban | Enoxaparin | 2.01 (0.18, 22.02) | 2.96 |
| 22 | Rivaroxaban | Nadroparin | 0.44 (0.10, 2.04) | 6.72 |
| Overall  (I^2^=12.9%, p=0.306) | NOACs | non-NOAC | 0.82 (0.54, 1.26) | 100.00 |

^a^ Weights are from random effects analysis

1. **Major VTE**

| Study | t1 | t2 | RR (95% CI) | ^a^Weigh (%) |
| --- | --- | --- | --- | --- |
| 1 | Rivaroxaban | Enoxaparin | 0.12 (0.04, 0.34) | 5.98 |
| 2 | Rivaroxaban | Enoxaparin | 0.12 (0.05, 0.29) | 7.00 |
| 3 | Rivaroxaban | Enoxaparin | 0.38 (0.18, 0.82) | 7.47 |
| 4 | Rivaroxaban | Enoxaparin | 0.58 (0.30, 1.16) | 7.94 |
| 6 | apixaban | Enoxaparin | 1.35 (0.72, 2.52) | 8.28 |
| 7 | apixaban | Enoxaparin | 0.50 (0.26, 0.97) | 8.06 |
| 8 | apixaban | Enoxaparin | 0.40 (0.19, 0.83) | 7.65 |
| 9 | dabigatran | Enoxaparin | 0.92 (0.61, 1.37) | 9.50 |
| 10 | dabigatran | Enoxaparin | 0.54 (0.31, 0.96) | 8.60 |
| 11 | dabigatran | Enoxaparin | 0.92 (0.52, 1.62) | 8.61 |
| 12 | dabigatran | Enoxaparin | 1.37 (0.76, 2.47) | 8.50 |
| 13 | dabigatran | Placo | 0.16 (0.03, 0.79) | 3.77 |
| 21 | Rivaroxaban | TB-402 | 1.00 (0.19, 5.43) | 3.47 |
| 22 | Rivaroxaban | Nadroparin | 0.32 (0.09, 1.06) | 5.16 |
| Overall  (I^2^=74.0%, p=0.000) | NOACs | non-NOAC | **0.52 (0.35, 0.76)** | 100.00 |

^a^ Weights are from random effects analysis

1. **Major bleeding**

| Study | t1 | t2 | RR (95% CI) | ^a^Weigh (%) |
| --- | --- | --- | --- | --- |
| 1 | Rivaroxaban | Enoxaparin | 3.02 (0.61, 14.95) | 4.33 |
| 2 | Rivaroxaban | Enoxaparin | 1.00 (0.06, 16.03) | 2.36 |
| 3 | Rivaroxaban | Enoxaparin | 1.19 (0.40, 3.53) | 5.57 |
| 4 | Rivaroxaban | Enoxaparin | 2.47 (0.78, 7.86) | 5.39 |
| 5 | Apixaban | Enoxaparin | 4.43 (0.24, 81.71) | 2.20 |
| 6 | Apixaban | Enoxaparin | 0.52 (0.25, 1.08) | 6.45 |
| 7 | Apixaban | Enoxaparin | 0.64 (0.28, 1.48) | 6.19 |
| 8 | Apixaban | Enoxaparin | 1.22 (0.66, 2.27) | 6.68 |
| 9 | Dabigatran | Enoxaparin | 1.06 (0.61, 1.84) | 6.81 |
| 10 | Dabigatran | Enoxaparin | 1.55 (0.67, 3.55) | 6.20 |
| 11 | Dabigatran | Enoxaparin | 1.06 (0.48, 2.33) | 6.30 |
| 12 | Dabigatran | Enoxaparin | 0.42 (0.18, 0.97) | 6.19 |
| 13 | Dabigatran | Placo | 1.46 (0.15, 13.88) | 3.07 |
| 14 | Betrixaban | Enoxaparin | 0.09 (0.00, 2.06) | 1.94 |
| 15 | Rivaroxaban | Aspirin | 0.62 (0.20, 1.90) | 5.50 |
| 16 | Edoxaban | Enoxaparin | 3.95 (0.44, 35.10) | 3.18 |
| 17 | Edoxaban | Enoxaparin | 0.33 (0.07, 1.62) | 4.34 |
| 21 | Rivaroxaban | TB-402 | 0.11 (0.01, 1.80) | 2.29 |
| 22 | Rivaroxaban | Nadroparin | 14.18 (7.67, 26.22) | 6.69 |
| 23 | Dabigatran | Enoxaparin | 1.50 (0.26, 8.55) | 4.02 |
| 24 | Rivaroxaban/ Dabigatran | Enoxaparin | 0.50 (0.03, 7.86) | 2.38 |
| 25 | Edoxaban | Enoxaparin | 1.51 (0.06, 36.65) | 1.93 |
| Overall  (I^2^=76.6%, p=0.000) | NOACs | non-NOAC | 1.14 (0.68, 1.91) | 100.00 |

^a^ Weights are from random effects analysis

1. **All bleeding**

| Study | t1 | t2 | RR (95% CI) | ^a^Weigh (%) |
| --- | --- | --- | --- | --- |
| 1 | Rivaroxaban | Enoxaparin | 1.02 (0.81, 1.29) | 10.93 |
| 2 | Rivaroxaban | Enoxaparin | 1.20 (0.88, 1.64) | 7.53 |
| 3 | Rivaroxaban | Enoxaparin | 1.02 (0.72, 1.44) | 6.41 |
| 4 | Rivaroxaban | Enoxaparin | 1.11 (0.90, 1.38) | 12.03 |
| 5 | Apixaban | Enoxaparin | 0.67 (0.28, 1.64) | 1.25 |
| 6 | Apixaban | Enoxaparin | 0.79 (0.60, 1.04) | 8.94 |
| 7 | Apixaban | Enoxaparin | 0.83 (0.64, 1.06) | 10.11 |
| 8 | Apixaban | Enoxaparin | 0.93 (0.81, 1.08) | 16.92 |
| 10 | Dabigatran | Enoxaparin | 1.17 (0.89, 1.55) | 8.80 |
| 13 | Dabigatran | Placo | 1.31 (0.66, 2.63) | 2.00 |
| 14 | Betrixaban | Enoxaparin | 0.17 (0.03, 0.97) | 0.33 |
| 15 | Rivaroxaban | Aspirin | 0.77 (0.41, 1.44) | 2.39 |
| 16 | Edoxaban | Enoxaparin | 1.67 (0.86, 3.25) | 2.15 |
| 17 | Edoxaban | Enoxaparin | 1.28 (0.91, 1.79) | 6.62 |
| 25 | Edoxaban | Enoxaparin | 0.90 (0.54, 1.47) | 3.60 |
| Overall  (I^2^=29.8%, p=0.132) | NOACs | non-NOAC | 1.00 (0.91, 1.11) | 100.00 |

^a^ Weights are from random effects analysis

1. **Clinically relevant nonmajor bleeding**

| Study | t1 | t2 | RR (95% CI) | ^a^Weigh (%) |
| --- | --- | --- | --- | --- |
| 1 | Rivaroxaban | Enoxaparin | 1.21 (0.85, 1.73) | 10.34 |
| 2 | Rivaroxaban | Enoxaparin | 1.22 (0.77, 1.92) | 6.96 |
| 3 | Rivaroxaban | Enoxaparin | 1.20 (0.73, 1.97) | 5.95 |
| 4 | Rivaroxaban | Enoxaparin | 1.29 (0.80, 2.06) | 6.55 |
| 6 | Apixaban | Enoxaparin | 0.74 (0.48, 1.15) | 7.57 |
| 7 | Apixaban | Enoxaparin | 0.76 (0.52, 1.12) | 9.13 |
| 8 | Apixaban | Enoxaparin | 0.91 (0.70, 1.17) | 16.69 |
| 9 | Dabigatran | Enoxaparin | 1.29 (0.90, 1.84) | 10.24 |
| 10 | Dabigatran | Enoxaparin | 1.14 (0.63, 2.07) | 4.36 |
| 11 | Dabigatran | Enoxaparin | 1.19 (0.82, 1.73) | 9.61 |
| 12 | Dabigatran | Enoxaparin | 1.03 (0.61, 1.72) | 5.60 |
| 13 | Dabigatran | Placo | 0.49 (0.10, 2.38) | 0.66 |
| 14 | Betrixaban | Enoxaparin | 0.25 (0.04, 1.74) | 0.45 |
| 16 | Edoxaban | Enoxaparin | 1.48 (0.73, 3.02) | 3.11 |
| 17 | Edoxaban | Enoxaparin | 1.18 (0.37, 3.84) | 1.19 |
| 21 | Rivaroxaban | TB-402 | 0.32 (0.10, 1.06) | 1.14 |
| 25 | Edoxaban | Enoxaparin | 0.50 (0.07, 3.49) | 0.44 |
| Overall  (I^2^=13.5%, p=0.296) | NOACs | non-NOAC | 1.04 (0.91, 1.18) | 100.00 |

^a^ Weights are from random effects analysis

1. **Ischemic stroke**

| Study | t1 | t2 | RR (95% CI) | ^a^Weigh (%) |
| --- | --- | --- | --- | --- |
| 1 | Rivaroxaban | Enoxaparin | 1.01 (0.20, 4.98) | 22.88 |
| 2 | Rivaroxaban | Enoxaparin | 2.01 (0.18, 22.12) | 10.17 |
| 3 | Rivaroxaban | Enoxaparin | 7.13 (0.37, 137.86) | 6.67 |
| 4 | Rivaroxaban | Enoxaparin | 1.48 (0.25, 8.86) | 18.31 |
| 5 | Apixaban | Enoxaparin | 2.46 (0.12, 50.92) | 6.37 |
| 6 | Apixaban | Enoxaparin | 0.20 (0.01, 4.16) | 6.35 |
| 7 | Apixaban | Enoxaparin | 5.00 (0.24, 104.13) | 6.35 |
| 8 | Apixaban | Enoxaparin | 0.20 (0.02, 1.71) | 12.70 |
| 22 | Rivaroxaban | Nadroparin | 4.00 (0.36, 43.92) | 10.19 |
| Overall  (I2=0.0%, p=0.465) | NOACs | non-NOAC | 1.31 (0.61, 2.81) | 100.00 |

^a^ Weights are from random effects analysis

1. **Myocardial infarction**

| Study | t1 | t2 | RR (95% CI) | ^a^Weigh (%) |
| --- | --- | --- | --- | --- |
| 1 | Rivaroxaban | Enoxaparin | 1.18 (0.40, 3.49) | 25.20 |
| 2 | Rivaroxaban | Enoxaparin | 1.34 (0.30, 5.97) | 13.37 |
| 3 | Rivaroxaban | Enoxaparin | 0.51 (0.05, 5.61) | 5.19 |
| 4 | Rivaroxaban | Enoxaparin | 0.20 (0.02, 1.69) | 6.49 |
| 5 | Apixaban | Enoxaparin | 3.44 (0.18, 66.25) | 3.42 |
| 6 | Apixaban | Enoxaparin | 0.40 (0.08, 2.06) | 11.13 |
| 7 | Apixaban | Enoxaparin | 1.00 (0.06, 15.98) | 3.89 |
| 8 | Apixaban | Enoxaparin | 2.24 (0.69, 7.27) | 21.58 |
| 10 | Dabigatran | Enoxaparin | 0.99 (0.06, 15.86) | 3.89 |
| 22 | Rivaroxaban | Nadroparin | 6.00 (0.63, 57.42) | 5.86 |
| Overall  (I2=0.0%, p=0.484) | NOACs | non-NOAC | 1.17 (0.68, 2.02) | 100.00 |

^a^ Weights are from random effects analysis

**Supplementary Table 4** SUCRA ranking of different NOACs and conventional anticoagulants

| **Treatments** | **SUCRA** | **PrBest** | **MeanRank** | **Treatments** | **SUCRA** | **PrBest** | **MeanRank** |
| --- | --- | --- | --- | --- | --- | --- | --- |
| **VTE** | | | | **Major bleeding** | | | |
| Rivaroxaban | **79.6** | 10.2 | 2.8 | Placo | 58.0 | 4.7 | 4.8 |
| Apixaban | 65.5 | 4.5 | 4.1 | Enoxaparin | 48.2 | 0.0 | 5.7 |
| Dabigatran | 28.5 | 0.0 | 7.4 | Nadroparin | **92.5** | 39.6 | 1.7 |
| Edoxaban | 75.5 | 19.5 | 3.2 | Aspirin | 18.2 | 0.0 | 8.4 |
| Betrixaban | 21.8 | 1.4 | 8.0 | TB-402 | 4.3 | 0.0 | 9.6 |
| Placo | 6.4 | 0.0 | 9.4 | Rivaroxaban | 26.9 | 0.0 | 7.6 |
| Enoxaparin | 34.4 | 0.0 | 6.9 | Apixaban | 60.3 | 0.0 | 4.6 |
| Nadroparin | 41.4 | 4.5 | 6.3 | Dabigatran | 49.4 | 0.0 | 5.5 |
| Aspirin | 77.6 | 36.8 | 3.0 | Edoxaban | 53.3 | 0.1 | 5.2 |
| TB-402 | 69.3 | 23.0 | 3.8 | Betrixaban | 88.9 | 55.6 | 2.0 |
| **All cause death** | | | | **All bleeding** | | | |
| Rivaroxaban | 58.4 | 0.3 | 4.7 | Rivaroxaban | 38.6 | 0.0 | 5.3 |
| Apixaban | 31.5 | 0.1 | 7.2 | Apixaban | 77.2 | 1.4 | 2.6 |
| Dabigatran | 31.8 | 0.3 | 7.1 | Dabigatran | 28.2 | 0.1 | 6.0 |
| Edoxaban | 50.8 | 8.5 | 5.4 | Edoxaban | 24.0 | 0.0 | 6.3 |
| Betrixaban | 67.9 | 31.8 | 3.9 | Betrixaban | **97.6** | 94.8 | 1.2 |
| Placo | 26.8 | 5.4 | 7.6 | Placo | 60.2 | 3.3 | 3.8 |
| Enoxaparin | 39.8 | 0.1 | 6.4 | Enoxaparin | 56.3 | 0.0 | 4.1 |
| Nadroparin | 41.3 | 0.4 | 6.3 | Aspirin | 17.8 | 0.3 | 6.8 |
| Aspirin | **84.4** | 33.7 | 2.4 | - | - | - | - |
| TB-402 | 67.3 | 19.5 | 3.9 | - | - | - | - |
| **DVT** | | | | **Clinically relevant nonmajor bleeding** | | | |
| Rivaroxaban | **88.8** | 39.3 | 1.9 | Rivaroxaban | 40.5 | 0.0 | 5.2 |
| Apixaban | 73.4 | 11.5 | 3.1 | Apixaban | 83.9 | 9.1 | 2.1 |
| Dabigatran | 36.5 | 0.1 | 6.1 | Dabigatran | 43.5 | 0.0 | 5.0 |
| Edoxaban | 71.8 | 23.4 | 3.3 | Edoxaban | 42.3 | 0.9 | 5.0 |
| Betrixaban | 28.8 | 4.2 | 6.7 | Betrixaban | **94.1** | 87.6 | 1.4 |
| Placo | 9.1 | 0.2 | 8.3 | Placo | 22.5 | 2.0 | 6.4 |
| Enoxaparin | 38.5 | 0.0 | 5.9 | Enoxaparin | 66.3 | 0.2 | 3.4 |
| Nadroparin | 52.0 | 15.0 | 4.8 | TB-402 | 6.8 | 0.1 | 7.5 |
| Aspirin | 51.0 | 6.4 | 4.9 | - | - | - | - |
| **PE** | | | | **Ischemic stroke** | | | |
| Rivaroxaban | 61.4 | 4.1 | 4.1 | Rivaroxaban | 21.8 | 0.7 | 4.1 |
| Apixaban | 46.2 | 2.9 | 5.3 | Apixaban | 64.3 | 22.4 | 2.4 |
| Dabigatran | 63.2 | 8.8 | 3.9 | Dabigatran | 46.4 | 27.2 | 3.1 |
| Edoxaban | 54.5 | 20.1 | 4.6 | Enoxaparin | 44.9 | 2.7 | 3.2 |
| Betrixaban | 42.5 | 16.7 | 5.6 | Nadroparin | **72.5** | 47.0 | 2.1 |
| Placo | 42.4 | 21.9 | 5.6 | - | - | - | - |
| Enoxaparin | 43.1 | 0.2 | 5.6 | - | - | - | - |
| Nadroparin | 31.2 | 4.1 | 6.5 | - | - | - | - |
| Aspirin | **65.5** | 21.2 | 3.8 | - | - | - | - |
| **Major VTE** | | | | **Myocardial infarction** | | | |
| Rivaroxaban | **89.9** | 45.0 | 1.6 | Rivaroxaban | 49.4 | 2.3 | 3.0 |
| Apixaban | 57.3 | 1.5 | 3.6 | Apixaban | 25.4 | 1.2 | 4.0 |
| Dabigatran | 41.4 | 0.0 | 4.5 | Dabigatran | 42.7 | 15.0 | 3.3 |
| Placo | 3.3 | 0.1 | 6.8 | Enoxaparin | 41.1 | 1.6 | 3.4 |
| Enoxaparin | 32.0 | 0.0 | 5.1 | Nadroparin | **91.3** | 79.9 | 1.3 |
| Nadroparin | 44.8 | 4.0 | 4.3 | - | - | - | - |
| TB-402 | 81.3 | 49.4 | 2.1 | - | - | - | - |

SUCRA: the surface under the cumulative ranking curve; Pr. Best: probability of being the best. Red bolded values are the most effective treatment (with the biggest SUCRA value). CRNMB, clinically relevant nonmajor bleeding.

**Supplementary Table 5** Forest plot results of direct analysis for different outcomes in subgroups

| **Comparison** | **Outcome** | **RR (CI 95%)** | **I^2^** | **p** |
| --- | --- | --- | --- | --- |
| ***THA*** | | | | |
| NOACs vs non-NOAC | VTE | **0.59(0.38, 0.92)** | 84.2% | 0.000 |
|  | All cause death | 0.90(0.44, 1.83) | 23.8% | 0.247 |
|  | DVT | **0.53(0.32, 0.88)** | 86.4% | 0.000 |
|  | PE | 0.68(0.36, 1.27) | 0.0% | 0.531 |
|  | Major VTE | **0.37(0.20, 0.70)** | 78.7% | 0.000 |
|  | Major bleeding | 1.41(0.58, 3.38) | 84.3% | 0.000 |
|  | All bleeding | 1.03(0.93, 1.14) | 3.8% | 0.397 |
|  | CRNMB | 1.07(0.89, 1.28) | 12.9% | 0.330 |
|  | Ischemic stroke | 1.03(0.32, 3.32) | 20.6% | 0.286 |
|  | Myocardial infarction | 1.67(0.87, 3.21) | 0.0% | 0.719 |
| ***TKA*** | | | | |
| NOACs vs non-NOAC | VTE | **0.75(0.59, 0.97)** | 86.5% | 0.000 |
|  | All cause death | 0.81(0.41, 1.62) | 0.0% | 0.609 |
|  | DVT | **0.73(0.57, 0.93)** | 87.2% | 0.000 |
|  | PE | 0.91(0.50, 1.66) | 22.8% | 0.241 |
|  | Major VTE | 0.71(0.46, 1.11) | 63.5% | 0.012 |
|  | Major bleeding | 0.85(0.56, 1.29) | 28.1% | 0.169 |
|  | All bleeding | 0.93(0.77, 1.13) | 43.3% | 0.090 |
|  | CRNMB | 1.00(0.82, 1.22) | 21.4% | 0.252 |
|  | Ischemic stroke | 1.81(0.57, 5.72) | 0.0% | 0.498 |
|  | Myocardial infarction | 0.51(0.19, 1.39) | 0.0% | 0.615 |
| ***Follow-up duration, d<60*** | | | | |
| NOACs vs non-NOAC | VTE | **0.54(0.42, 0.70)** | 64.8% | 0.001 |
|  | All cause death | 0.71(0.42, 1.21) | 0.0% | 0.444 |
|  | DVT | **0.54(0.41, 0.71)** | 69.3% | 0.000 |
|  | PE | 0.60(0.29, 1.24) | 6.6% | 0.379 |
|  | Major VTE | **0.28(0.16, 0.51)** | 56.5% | 0.032 |
|  | Major bleeding | 1.43(0.55, 3.72) | 74.6% | 0.000 |
|  | All bleeding | 1.10(0.97, 1.24) | 5.7% | 0.389 |
|  | CRNMB | 1.14(0.93, 1.41) | 5.1% | 0.394 |
|  | Ischemic stroke | 1.89(0.78, 4.58) | 0.0% | 0.870 |
|  | Myocardial infarction | 1.15(0.53, 2.52) | 11.6% | 0.341 |
| ***Follow-up duration, d≥60*** | | | | |
| NOACs vs non-NOAC | VTE | 0.87(0.66, 1.14) | 88.3% | 0.000 |
|  | All cause death | 1.55(0.70, 3.42) | 9.3% | 0.358 |
|  | DVT | 0.83(0.63, 1.09) | 89.4% | 0.000 |
|  | PE | 0.97(0.58, 1.62) | 15.3% | 0.310 |
|  | Major VTE | 0.80(0.57, 1.11) | 56.6% | 0.032 |
|  | Major bleeding | 0.87(0.65, 1.16) | 17.7% | 0.285 |
|  | All bleeding | 0.91(0.80, 1.04) | 23.5% | 0.264 |
|  | CRNMB | 0.98(0.83, 1.15) | 16.9% | 0.301 |
|  | Ischemic stroke | 0.50(0.07, 3.65) | 38.4% | 0.197 |
|  | Myocardial infarction | 1.19(0.51, 2.82) | 0.0% | 0.415 |

The cells contain the relative risk (RR), 95% confidence interval (CI) of the treatment comparison. Red bolded values are statistically significant. CRNMB, clinically relevant nonmajor bleeding. I^2^ (I-squared test result) and p (Q statistic) were used to assess the between-study heterogeneity.

**Supplementary Table 6** Network meta-analysis of the subgroups

1. **THA**

| **Outcome** | **Comparison** | **RR (CI 95%)** | **Outcome** | **Comparison** | **RR (CI 95%)** |
| --- | --- | --- | --- | --- | --- |
| **VTE** | Apixaban vs Rivaroxaban | 1.23(0.26, 5.79) | **Major bleeding** | Apixaban vs Rivaroxaban | 0.53(0.12, 2.42) |
|  | Dabigatran vs Rivaroxaban | 3.31(0.66, 16.66) |  | Dabigatran vs Rivaroxaban | 0.52(0.12, 2.23) |
|  | Edoxaban vs Rivaroxaban | 1.51(0.25, 9.09) |  | Edoxaban vs Rivaroxaban | 0.19(0.03, 1.42) |
|  | Dabigatran vs Apixaban | 2.69(0.80, 8.99) |  | Dabigatran vs Apixaban | 0.97(0.45, 2.11) |
|  | Edoxaban vs Apixaban | 1.23(0.29, 5.16) |  | Edoxaban vs Apixaban | 0.37(0.08, 1.73) |
|  | Edoxaban vs Dabigatran | 0.46(0.10, 2.06) |  | Edoxaban vs Dabigatran | 0.38(0.08, 1.68) |
| **All cause death** | Apixaban vs Rivaroxaban | 3.95(0.22, 69.36) | **All bleeding** | Apixaban vs Rivaroxaban | 0.86(0.68, 1.09) |
|  | Dabigatran vs Rivaroxaban | 1.93(0.11, 34.27) |  | Dabigatran vs Rivaroxaban | 1.08(0.77, 1.52) |
|  | Edoxaban vs Rivaroxaban | 0.80(0.01, 76.43) |  | Edoxaban vs Rivaroxaban | 1.05(0.75, 1.48) |
|  | Dabigatran vs Apixaban | 0.49(0.02, 15.36) |  | Dabigatran vs Apixaban | 1.26(0.92, 1.72) |
|  | Edoxaban vs Apixaban | 0.20(0.00, 28.31) |  | Edoxaban vs Apixaban | 1.22(0.89, 1.67) |
|  | Edoxaban vs Dabigatran | 0.41(0.00, 58.35) |  | Edoxaban vs Dabigatran | 0.97(0.65, 1.44) |
| **DVT** | Apixaban vs Rivaroxaban | 0.88(0.17, 4.58) | **CRNMB** | Apixaban vs Rivaroxaban | 0.75(0.51, 1.09) |
|  | Dabigatran vs Rivaroxaban | 2.62(0.72, 9.47) |  | Dabigatran vs Rivaroxaban | 1.03(0.68, 1.56) |
|  | Edoxaban vs Rivaroxaban | 2.27(0.27, 18.86) |  | Edoxaban vs Rivaroxaban | 0.77(0.27, 2.20) |
|  | Dabigatran vs Apixaban | 2.98(0.53, 16.64) |  | Dabigatran vs Apixaban | 1.38(0.92, 2.05) |
|  | Edoxaban vs Apixaban | 2.58(0.23, 28.65) |  | Edoxaban vs Apixaban | 1.04(0.37, 2.93) |
|  | Edoxaban vs Dabigatran | 0.87(0.10, 7.62) |  | Edoxaban vs Dabigatran | 0.75(0.26, 2.16) |
| **PE** | Apixaban vs Rivaroxaban | 0.27(0.04, 1.84) | **Ischemic stroke** | Apixaban vs Rivaroxaban | 0.16(0.01, 2.00) |
|  | Dabigatran vs Rivaroxaban | 0.67(0.11, 4.32) |  | Dabigatran vs Rivaroxaban | 0.80(0.01, 50.03) |
|  | Edoxaban vs Rivaroxaban | 0.57(0.03, 12.52) |  | Dabigatran vs Apixaban | 4.98(0.06, 434.44) |
|  | Dabigatran vs Apixaban | 2.50(0.38, 16.62) |  | - | - |
|  | Edoxaban vs Apixaban | 2.12(0.09, 47.66) |  | - | - |
|  | Edoxaban vs Dabigatran | 0.85(0.44, 18.26) |  | - | - |
| **Major VTE** | Apixaban vs Rivaroxaban | **3.25(1.10, 9.66)** | **Myocardial infarction** | Apixaban vs Rivaroxaban | 1.82(0.42, 7.93) |
|  | Dabigatran vs Rivaroxaban | **6.07(2.63, 14.05)** |  | Dabigatran vs Rivaroxaban | 0.81(0.04, 14.79) |
|  | Dabigatran vs Apixaban | 1.87(0.73, 4.77) |  | Dabigatran vs Apixaban | 0.44(0.02, 8.98) |

The cells contain the relative risk (RR), 95% confidence interval (CI) of the treatment comparison. Red bolded values are statistically significant.

1. **TKA**

| **Outcome** | **Comparison** | **RR (CI 95%)** | **Outcome** | **Comparison** | **RR (CI 95%)** |
| --- | --- | --- | --- | --- | --- |
| **VTE** | Apixaban vs Rivaroxaban | 0.79(0.48, 1.29) | **Major bleeding** | Apixaban vs Rivaroxaban | 0.37(0.13, 1.04) |
|  | Dabigatran vs Rivaroxaban | 1.53(0.97, 2.42) |  | Dabigatran vs Rivaroxaban | 0.44(0.16, 1.23) |
|  | Edoxaban vs Rivaroxaban | 0.72(0.34, 1.50) |  | Edoxaban vs Rivaroxaban | 2.35(0.22, 25.03) |
|  | Betrixaban vs Rivaroxaban | 2.04(0.65, 6.41) |  | Betrixaban vs Rivaroxaban | 0.05(0.00, 1.39) |
|  | Dabigatran vs Apixaban | **1.95(1.18, 3.21)** |  | Dabigatran vs Apixaban | 1.19(0.52, 2.77) |
|  | Edoxaban vs Apixaban | 0.91(0.43, 1.95) |  | Edoxaban vs Apixaban | 6.34(0.64, 63.04) |
|  | Betrixaban vs Apixaban | 2.60(0.81, 8.29) |  | Betrixaban vs Apixaban | 0.14(0.01, 3.57) |
|  | Edoxaban vs Dabigatran | **0.47(0.22, 0.98)** |  | Edoxaban vs Dabigatran | 5.31(0.54, 52.54) |
|  | Betrixaban vs Dabigatran | 1.33(0.42, 4.20) |  | Betrixaban vs Dabigatran | 0.11(0.00, 2.98) |
|  | Betrixaban vs Edoxaban | 2.85(0.79, 10.28) |  | Betrixaban vs Edoxaban | 0.02(0.00, 1.06) |
| **All cause death** | Apixaban vs Rivaroxaban | 1.53(0.16, 14.90) | **All bleeding** | Apixaban vs Rivaroxaban | **0.74(0.57, 0.95)** |
|  | Dabigatran vs Rivaroxaban | 2.17(0.20, 23.60) |  | Edoxaban vs Rivaroxaban | 1.54(0.77, 3.07) |
|  | Edoxaban vs Rivaroxaban | 1.60(0.02, 118.99) |  | Betrixaban vs Rivaroxaban | **0.15(0.03, 0.90)** |
|  | Betrixaban vs Rivaroxaban | 0.41(0.01, 30.55) |  | Edoxaban vs Apixaban | **2.08(1.04, 4.16)** |
|  | Dabigatran vs Apixaban | 1.42(0.15, 13.20) |  | Betrixaban vs Apixaban | 0.21(0.04, 1.22) |
|  | Edoxaban vs Apixaban | 1.04(0.02, 72.28) |  | Betrixaban vs Edoxaban | **0.10(0.02, 0.66)** |
|  | Betrixaban vs Apixaban | 0.27(0.00, 18.55) |  | - | - |
|  | Edoxaban vs Dabigatran | 0.74(0.01, 59.40) |  | - | - |
|  | Betrixaban vs Dabigatran | 0.19(0.00, 15.26) |  | - | - |
|  | Betrixaban vs Edoxaban | 0.26(0.00, 75.25) |  | - | - |
| **DVT** | Apixaban vs Rivaroxaban | 1.23(0.72, 2.10) | **CRNMB** | Apixaban vs Rivaroxaban | **0.60(0.39, 0.95)** |
|  | Dabigatran vs Rivaroxaban | **1.93(1.12, 3.32)** |  | Dabigatran vs Rivaroxaban | 0.91(0.58, 1.44) |
|  | Edoxaban vs Rivaroxaban | 0.89(0.40, 2.00) |  | Edoxaban vs Rivaroxaban | 1.19(0.54, 2.62) |
|  | Betrixaban vs Rivaroxaban | 2.34(0.71, 7.71) |  | Betrixaban vs Rivaroxaban | 0.20(0.03, 1.44) |
|  | Dabigatran vs Apixaban | 1.57(0.95, 2.59) |  | Dabigatran vs Apixaban | 1.51(0.99, 2.29) |
|  | Edoxaban vs Apixaban | 0.73(0.33, 1.5) |  | Edoxaban vs Apixaban | 1.97(0.91, 4.24) |
|  | Betrixaban vs Apixaban | 1.90(0.59, 6.16) |  | Betrixaban vs Apixaban | 0.33(0.05, 2.36) |
|  | Edoxaban vs Dabigatran | 0.46(0.21, 1.02) |  | Edoxaban vs Dabigatran | 1.30(0.60, 2.83) |
|  | Betrixaban vs Dabigatran | 1.21(0.37, 3.95) |  | Betrixaban vs Dabigatran | 0.22(0.03, 1.57) |
|  | Betrixaban vs Edoxaban | 2.61(0.70, 9.78) |  | Betrixaban vs Edoxaban | 0.17(0.02, 1.33) |
| **PE** | Apixaban vs Rivaroxaban | 2.94(0.85, 10.18) | **Ischemic stroke** | Apixaban vs Rivaroxaban | 0.59(0.05, 7.06) |
|  | Dabigatran vs Rivaroxaban | 1.22(0.28, 5.37) |  | - | - |
|  | Edoxaban vs Rivaroxaban | 1.98(0.03, 114.11) |  | - | - |
|  | Betrixaban vs Rivaroxaban | 2.57(0.11, 62.61) |  | - | - |
|  | Dabigatran vs Apixaban | 0.42(0.12, 1.45) |  | - | - |
|  | Edoxaban vs Apixaban | 0.67(0.01, 35.90) |  | - | - |
|  | Betrixaban vs Apixaban | 0.87(0.04, 19.27) |  | - | - |
|  | Edoxaban vs Dabigatran | 1.62(0.03, 93.40) |  | - | - |
|  | Betrixaban vs Dabigatran | 2.10(0.09, 51.27) |  | - | - |
|  | Betrixaban vs Edoxaban | 1.30(0.01, 182.04) |  | - | - |
| **Major VTE** | Apixaban vs Rivaroxaban | 1.74(0.67, 4.51) | **Myocardial infarction** | Apixaban vs Rivaroxaban | 2.40(0.31, 18.54) |
|  | Dabigatran vs Rivaroxaban | 2.34(0.93, 5.93) |  | - | - |
|  | Dabigatran vs Apixaban | 1.34(0.55, 3.30) |  | - | - |

The cells contain the relative risk (RR), 95% confidence interval (CI) of the treatment comparison. Red bolded values are statistically significant.

1. **Follow-up duration, d<60**

| **Outcome** | **Comparison** | **RR (CI 95%)** | **Outcome** | **Comparison** | **RR (CI 95%)** |
| --- | --- | --- | --- | --- | --- |
| **VTE** | Apixaban vs Rivaroxaban | 0.79(0.22, 2.86) | **Major bleeding** | Apixaban vs Rivaroxaban | 2.64(0.13, 52.57) |
|  | Edoxaban vs Rivaroxaban | 0.84(0.20, 3.47) |  | Dabigatran vs Rivaroxaban | 0.89(0.10, 7.65) |
|  | Betrixaban vs Rivaroxaban | 2.49(0.39, 16.05) |  | Edoxaban vs Rivaroxaban | 0.51(0.13, 2.00) |
|  | Edoxaban vs Apixaban | 1.06(0.42, 2.69) |  | Betrixaban vs Rivaroxaban | 0.05(0.00, 1.32) |
|  | Betrixaban vs Apixaban | 3.17(0.69, 14.45) |  | Dabigatran vs Apixaban | 0.34(0.01, 12.23) |
|  | Betrixaban vs Edoxaban | 2.98(0.58, 15.24) |  | Edoxaban vs Apixaban | 0.19(0.01, 4.50) |
|  | - | - |  | Betrixaban vs Apixaban | 0.02(0.00, 1.44) |
|  | - | **-** |  | Edoxaban vs Dabigatran | 0.57(0.05, 6.41) |
|  | - | - |  | Betrixaban vs Dabigatran | 0.06(0.00, 2.59) |
|  | - | - |  | Betrixaban vs Edoxaban | 0.10(0.00, 2.99) |
| **All cause death** | Apixaban vs Rivaroxaban | 2.30(0.08, 69.71) | **All bleeding** | Apixaban vs Rivaroxaban | 0.62(0.25, 1.53) |
|  | Edoxaban vs Rivaroxaban | 1.10(0.06, 21.29) |  | Edoxaban vs Rivaroxaban | 1.11(0.83, 1.49) |
|  | Betrixaban vs Rivaroxaban | 0.40(0.01, 23.70) |  | Betrixaban vs Rivaroxaban | **0.15(0.03, 0.90)** |
|  | Edoxaban vs Apixaban | 0.48(0.01, 36.26) |  | Edoxaban vs Apixaban | 1.79(0.71, 4.52) |
|  | Betrixaban vs Apixaban | 0.17(0.00, 30.25) |  | Betrixaban vs Apixaban | 0.25(0.03, 1.78) |
|  | Betrixaban vs Edoxaban | 0.36(0.00, 47.78) |  | Betrixaban vs Edoxaban | **0.14(0.02, 0.82)** |
| **DVT** | Apixaban vs Rivaroxaban | 1.52(0.31, 7.51) | **CRNMB** | Edoxaban vs Rivaroxaban | 1.04(0.56, 1.93) |
|  | Edoxaban vs Rivaroxaban | 1.37(0.37, 5.07) |  | Betrixaban vs Rivaroxaban | 0.21(0.03, 1.43) |
|  | Betrixaban vs Rivaroxaban | 3.06(0.51, 18.19) |  | Betrixaban vs Edoxaban | 0.20(0.03, 1.49) |
|  | Edoxaban vs Apixaban | 0.90(0.14, 5.82) |  | - | **-** |
|  | Betrixaban vs Apixaban | 2.01(0.22, 18.62) |  | - | - |
|  | Betrixaban vs Edoxaban | 2.23(0.30, 16.86) |  | - | - |
| **PE** | Apixaban vs Rivaroxaban | 0.14(0.01, 3.20) | **Ischemic stroke** | Apixaban vs Rivaroxaban | 1.53(0.06, 37.25) |
|  | Edoxaban vs Rivaroxaban | 1.11(0.10, 12.27) |  | - | - |
|  | Betrixaban vs Rivaroxaban | 1.80(0.08, 41.08) |  | - | - |
|  | Edoxaban vs Apixaban | 8.01(0.18, 350.99) | **Myocardial infarction** | Apixaban vs Rivaroxaban | 3.89(0.18, 82.52) |
|  | Betrixaban vs Apixaban | 13.00(0.18, 936.20) |  | - | - |
|  | Betrixaban vs Edoxaban | 1.62(0.04, 70.48) |  | - | - |

The cells contain the relative risk (RR), 95% confidence interval (CI) of the treatment comparison. Red bolded values are statistically significant.

1. **Follow-up duration, d≥60**

| **Outcome** | **Comparison** | **RR (CI 95%)** | **Outcome** | **Comparison** | **RR (CI 95%)** |
| --- | --- | --- | --- | --- | --- |
| **VTE** | Dabigatran vs Apixaban | **1.80(1.07, 3.04)** | **Major bleeding** | Dabigatran vs Apixaban | 1.25(0.62, 2.51) |
| **All cause death** | Dabigatran vs Apixaban | 1.03(0.17, 6.21) | **All bleeding** | Dabigatran vs Apixaban | 1.33(0.98, 1.79) |
| **DVT** | Dabigatran vs Apixaban | **1.76(1.09, 2.84)** | **CRNMB** | Dabigatran vs Apixaban | **1.42(1.07, 1.90)** |
| **PE** | Dabigatran vs Apixaban | 0.60(0.17, 2.16) | **Ischemic stroke** | Dabigatran vs Apixaban | 1.99(0.02, 258.39) |
| **Major VTE** | Dabigatran vs Apixaban | 1.34(0.64, 2.78) | **Myocardial infarction** | Dabigatran vs Apixaban | 0.92(0.03, 25.65) |

The cells contain the relative risk (RR), 95% confidence interval (CI) of the treatment comparison. Red bolded values are statistically significant.

**Supplementary Table 7** Results of node-splitting models.

| **Items** | **Comparison** | **Direct Coef** | **Indirect Coef** | **Difference Coef** | **P-value** |
| --- | --- | --- | --- | --- | --- |
| **DVT** | Apixaban, Enoxaparin | 0.51 | 0.03 | 0.48 | 0.98 |
|  | Aspirin, Rivaroxaban | -0.61 | -0.49 | -0.11 | 0.99 |
|  | Betrixaban, Enoxaparin | -0.32 | 1.22 | -1.55 | 0.99 |
|  | Dabigatran, Enoxaparin | -0.05 | 0.88 | -0.93 | 0.98 |
|  | Dabigatran, Placo | 0.72 | -0.98 | 1.70 | 0.98 |
|  | Edoxaban, Enoxaparin | 0.51 | 1.05 | -0.54 | 0.99 |
|  | Enoxaparin, Rivaroxaban | -0.78 | -0.89 | 0.113 | 0.99 |
|  | Nadroparin, Rivaroxaban | -0.59 | -0.28 | -0.31 | 0.99 |
| **PE** | Apixaban, Enoxaparin | 0.03 | 0.04 | -0.02 | 1.00 |
|  | Aspirin, Rivaroxaban | 0.18 | -0.59 | 0.77 | 0.99 |
|  | Betrixaban, Enoxaparin | -0.24 | 0.21 | -0.45 | 0.99 |
|  | Dabigatran, Enoxaparin | 0.36 | -0.12 | 0.24 | 0.99 |
|  | Dabigatran, Placo | 0.72 | -0.80 | -0.08 | 1.00 |
|  | Edoxaban, Enoxaparin | 0.23 | -0.12 | 0.25 | 1.00 |
|  | Enoxaparin, Rivaroxaban | -0.32 | -0.40 | -0.07 | 1.00 |
|  | Nadroparin, Rivaroxaban | -0.81 | -0.20 | -0.61 | 0.99 |
| **Major VTE** | Apixaban, Enoxaparin | 0.42 | 0.17 | 0.25 | 0.99 |
|  | Dabigatran, Enoxaparin | 0.12 | 1.10 | -0.98 | 0.99 |
|  | Dabigatran, Placo | 1.81 | -0.75 | 2.57 | 0.99 |
|  | Enoxaparin, Rivaroxaban | -1.35 | -1.13 | -0.20 | 0.99 |
|  | Nadroparin, Rivaroxaban | -1.15 | -1.22 | 0.07 | 1.00 |
|  | Rivaroxaban, TB-402 | -0.00 | 1.85 | -1.86 | 0.99 |
| **Major bleeding** | Apixaban, Enoxaparin | 0.20 | 0.10 | 0.10 | 0.99 |
|  | Betrixaban, Enoxaparin | 2.46 | 0.26 | 2.20 | 0.99 |
|  | Dabigatran, Enoxaparin | 0.03 | -3.39 | 3.42 | 0.28 |
|  | Dabigatran, Placo | -0.38 | -0.53 | 0.16 | 1.00 |
|  | Dabigatran, Rivaroxaban | -0.70 | 0.60 | -1.3 | 0.412 |
|  | Enoxaparin, Rivaroxaban | 0.51 | -0.36 | 0.88 | 0.695 |
|  | Enoxaparin, Edoxaban | -0.13 | -0.19 | 0.06 | 1.00 |
|  | Nadroparin, Rivaroxaban | 2.65 | 2.12 | 0.53 | 0.99 |
|  | Rivaroxaban, Aspirin | 0.47 | -1.46 | 1.94 | 0.99 |
|  | Rivaroxaban, TB-402 | 2.25 | -2.95 | 5.20 | 0.99 |

**Supplementary Table 8** Sensitivity analysis

| **Outcome** | **Study omitted** | **RR (CI 95%)** | **Outcome** | **Study omitted** | **RR (CI 95%)** |
| --- | --- | --- | --- | --- | --- |
| **DVT** | Eriksson 2008 | 0.69 (0.56, 0.86) | **PE** | Eriksson 2008 | 0.85 (0.59, 1.22) |
|  | Kakker 2008 | 0.70 (0.57, 0.86) |  | Kakker 2008 | 0.91 (0.63, 1.31) |
|  | Lassen 2008 | 0.67 (0.54, 0.84) |  | Lassen 2008 | 0.91 (0.64, 1.30) |
|  | Turpie 2009 | 0.65 (0.52, 0.82) |  | Turpie 2009 | 0.90 (0.60, 1.34) |
|  | Lassen 2007 | 0.66 (0.52, 0.82) |  | Lassen 2007 | 0.92 (0.64, 1.30) |
|  | Lassen 2009 | 0.64 (0.51, 0.81) |  | Lassen 2009 | 0.79 (0.53, 1.17) |
|  | Lassen 2010 | 0.66 (0.53, 0.84) |  | Lassen 2010 | 0.85 (0.60, 1.20) |
|  | Lassen 2010 | 0.69 (0.55, 0.85) |  | Lassen 2010 | 0.96 (0.67, 1.37) |
|  | Eriksson 2007 | 0.64 (0.51, 0.80) |  | Eriksson 2007 | 0.85 (0.57, 1.28) |
|  | Eriksson 2011 | 0.65 (0.51, 0.81) |  | Eriksson 2011 | 0.88 (0.60, 1.29) |
|  | Eriksson 2007 | 0.64 (0.50, 0.81) |  | Eriksson 2007 | 0.87 (0.59, 1.29) |
|  | Ginsberg 2009 | 0.63 (0.51, 0.78) |  | Ginsberg 2009 | 0.90 (0.60, 1.34) |
|  | Fuji 2010 | 0.67 (0.54, 0.84) |  | Turpie 2009 | 0.86 (0.58, 1.27) |
|  | Turpie 2009 | 0.64 (0.52, 0.80) |  | Anderson 2018 | 0.84 (0.56, 1.26) |
|  | Anderson 2018 | 0.65 (0.52, 0.82) |  | Kim 2016 | 0.85 (0.58, 1.25) |
|  | Fuji 2014 | 0.67 (0.53, 0.83) |  | Jiang 2014 | 0.82 (0.54, 1.26) |
|  | Kim 2016 | 0.64 (0.51, 0.81) |  | Jiang 2018 | 0.91 (0.62, 1.33) |
|  | Jiang 2014 | 0.65 (0.52, 0.81) |  | - | **-** |
|  | Zou 2014 | 0.67 (0.54, 0.84) |  | - | - |
|  | Jiang 2018 | 0.66 (0.53, 0.83) |  | - | - |
|  | Fuji 2014 | 0.66 (0.53, 0.82) |  | - | - |
| **Major VTE** | Eriksson 2008 | 0.57(0.40, 0.83) | **Major bleeding** | Eriksson 2008 | 1.09 (0.64, 1.85) |
|  | Kakker 2008 | 0.59(0.42, 0.83) |  | Kakker 2008 | 1.14 (0.67, 1.93) |
|  | Lassen 2008 | 0.53(0.35, 0.79) |  | Lassen 2008 | 1.13 (0.66, 1.95) |
|  | Turpie 2009 | 0.51(0.34, 0.77) |  | Turpie 2009 | 1.09 (0.63, 1.86) |
|  | Lassen 2009 | 0.48(0.32, 0.71) |  | Lassen 2007 | 1.10 (0.65, 1.87) |
|  | Lassen 2010 | 0.52(0.34, 0.78) |  | Lassen 2009 | 1.20 (0.70, 2.05) |
|  | Lassen 2010 | 0.53(0.35, 0.79) |  | Lassen 2010 | 1.18 (0.69, 2.03) |
|  | Eriksson 2007 | 0.48(0.32, 0.74) |  | Lassen 2010 | 1.13 (0.64, 1.98) |
|  | Eriksson 2011 | 0.51(0.34, 0.78) |  | Eriksson 2007 | 1.14 (0.64, 2.01) |
|  | Eriksson 2007 | 0.49(0.32, 0.74) |  | Eriksson 2011 | 1.11 (0.64, 1.93) |
|  | Ginsberg 2009 | 0.48(0.32, 0.70) |  | Eriksson 2007 | 1.14 (0.66, 1.98) |
|  | Fuji 2010 | 0.54(0.37, 0.80) |  | Ginsberg 2009 | 1.22 (0.72, 2.07) |
|  | Verhamme 2013 | 0.50(0.34, 0.75) |  | Fuji 2010 | 1.13 (0.66, 1.92) |
|  | Jiang 2018 | 0.53(0.36, 0.79) |  | Turpie 2009 | 1.20 (0.71, 2.01) |
|  | - | **-** |  | Anderson 2018 | 1.18 (0.69, 2.02) |
|  | - | - |  | Fuji 2014 | 1.09 (0.64, 1.85) |
|  | - | - |  | Fuji 2015 | 1.21 (0.71, 2.04) |
|  | - | **-** |  | Verhamme 2013 | 1.20 (0.72, 2.02) |
|  | - | - |  | Jiang 2018 | 0.94 (0.71, 1.24) |
|  | - | **-** |  | Mirdamadi 2014 | 1.12 (0.66, 1.92) |
|  | - | - |  | Özler 2015 | 1.16 (0.69, 1.96) |
|  | - | - |  | Fuji 2014 | 1.13 (0.67, 1.91) |

RR: relative risk; CI: confidence interval
